# A database overview of metal-coordination distances in metalloproteins

**DOI:** 10.1107/S2059798324003152

**Published:** 2024-04-29

**Authors:** Milana Bazayeva, Claudia Andreini, Antonio Rosato

**Affiliations:** a Department of Chemistry, University of Florence, Via della Lastruccia 3, 50019 Sesto Fiorentino, Italy; b Magnetic Resonance Center (CERM), University of Florence, Via Luigi Sacconi 6, 50019 Sesto Fiorentino, Italy; c Consorzio Interuniversitario di Risonanze Magnetiche di Metallo Proteine, Via Luigi Sacconi 6, 50019 Sesto Fiorentino, Italy; University of Oxford, United Kingdom

**Keywords:** alkali metals, alkaline-earth metals, transition metals, carboxylates, bidentate coordination, monodentate coordination, metalloproteins, metal-binding sites, bioinorganic chemistry, carboxylate coordination, carboxylate shift

## Abstract

Metalloproteins are ubiquitous in all living organisms and take part in a very wide range of biological processes. For this reason, their experimental characterization is crucial to obtain improved knowledge of their structure and biological functions. The three-dimensional structure represents highly relevant information since it provides insight into the interaction between the metal ion(s) and the protein fold. Such interactions determine the chemical reactivity of the bound metal. The available PDB structures can contain errors due to experimental factors such as poor resolution and radiation damage. A lack of use of distance restraints during the refinement and validation process also impacts the structure quality. Here, the aim was to obtain a thorough overview of the distribution of the distances between metal ions and their donor atoms through the statistical analysis of a data set based on more than 115 000 metal-binding sites in proteins. This analysis not only produced reference data that can be used by experimentalists to support the structure-determination process, for example as refinement restraints, but also resulted in an improved insight into how protein coordination occurs for different metals and the nature of their binding interactions. In particular, the features of carboxylate coordination were inspected, which is the only type of interaction that is commonly present for nearly all metals.

## Abstract 2

Through the analysis of more than 115 000 metal-binding sites in metalloproteins, experimentalists are offered useful reference data that improve the understanding of metal-binding interactions and protein coordination. Carboxylate coordination, which is common for a variety of metals, is especially highlighted.

## 1. Introduction

Metalloproteins (MPs) are a heterogeneous class of proteins with a metal ion as an integral part of their 3D structure. Their pervasive presence in all kingdoms of life underscores their fundamental roles in many biological processes, such as catalysis, electron transfer and oxygen transportation. Their importance is also reflected by the number of structures in the Protein Data Bank (PDB) that bear a metal cofactor (40%; Berman *et al.*, 2003 ▸; Andreini *et al.*, 2013 ▸; Putignano *et al.*, 2018 ▸). Within the protein architecture, metal-binding sites can be present as part of a catalytic site as well as participate in maintaining the protein structure, acting as a stabilizing agent (Harding, 2004 ▸).

The identification and modeling of metal centers during the determination of the 3D structures of biological macromolecules is not a trivial task. The chemical, crystallographic, biological and experimental aspects of the system under investigation must be taken into account (Touw *et al.*, 2016 ▸). The experimentally available data always come with uncertainties regarding the structural features that depend on the sample preparation (Németh *et al.*, 2014 ▸; Foster *et al.*, 2022 ▸), the data-collection protocol (McPherson, 2017 ▸) and the quality of the diffraction data, for example the resolution obtained (Zheng *et al.*, 2014 ▸). Since not all refinement programs apply geometric restrictions between the metal ion and its ligands by default, errors may occur during model generation and refinement of metal-binding sites (Zheng *et al.*, 2008 ▸; Roversi & Tronrud, 2021 ▸). This is particularly important for low-resolution structures (Touw *et al.*, 2016 ▸; Nicholls *et al.*, 2021 ▸). Indeed, several reports are available in the literature that describe inaccuracies observed in crystallographic structure models in the PDB at metal-binding sites (Zheng *et al.*, 2008 ▸, 2014 ▸; Raczynska *et al.*, 2016 ▸; Yao *et al.*, 2017 ▸), which can be as significant as an incorrect identification of the metal contained in the site (Grime *et al.*, 2020 ▸). It is often possible to reliably assign the type and/or position of metal ions through specific experiments (Garcia *et al.*, 2006 ▸), such as X-ray absorption near-edge structure (XANES; Ascone & Strange, 2009 ▸), extended X-ray absorption fine structure (EXAFS; Arcovito & della Longa, 2012 ▸; Hummer & Rompel, 2013 ▸), particle-induced X-ray emission (PIXE; Grime *et al.*, 2020 ▸) and the comparison of anomalous density maps (Bowman *et al.*, 2016 ▸). In the absence of such specific measurements, structure-validation tools (Gore *et al.*, 2017 ▸) may be of help. However, the validation of small-molecule ligands in crystallographic structures is an area of application that is still under significant development (Smart *et al.*, 2018 ▸). The latter remark also applies to metal ions (Handing *et al.*, 2018 ▸). In this respect, the *CheckMyMetal* (*CMM*) validation server is particularly relevant (Zheng *et al.*, 2014 ▸). Since its launch in 2012, *CMM* has been used in the validation of more than 130 000 protein structures (Gucwa *et al.*, 2023 ▸). Among other features, *CMM* outputs a score-ranked list of suggested metals for each metal-binding site, thereby facilitating the identification of erroneously assigned metal types.

Numerous databases exist that address MPs either in general or some specific aspects of their chemistry and biology (Zhang & Zheng, 2020 ▸; Andreini & Rosato, 2022 ▸). Of particular relevance for this work are MetalPDB (Andreini *et al.*, 2013 ▸; Putignano *et al.*, 2018 ▸) and MESPEUS (Lin *et al.*, 2024 ▸; Hsin *et al.*, 2008 ▸), both of which address the structural and functional properties of all metal-binding sites present in the PDB. The latter mostly focuses on the geometry of metal-binding sites; its web interface allows the user to search for metal sites using several options, including combinations of different metals, donor types and structure resolutions. The statistics of the retrieved metal–donor distances and coordination geometries can be visualized as a set of histograms or downloaded for further analysis with the user’s own tools. MetalPDB also emphasizes functional aspects by defining a minimal functional site around the metal ion for all metalloproteins of known 3D structure. In this work, we exploited the information on the metal-coordination environment that has already been calculated in the latter database (Andreini *et al.*, 2013 ▸; Putignano *et al.*, 2018 ▸).

The aim of this study is to deliver reliable information on the optimal coordination distances for most of the biologically relevant metals. To this end, we performed an extensive statistical analysis of the data in MetalPDB and compared our main observations with the corresponding work by Harding (2001 ▸, 2004 ▸, 2006 ▸) based on an analysis of the Cambridge Structural Database (CSD) and the PDB, along with statistics extracted from MESPEUS. In addition to the investigation of the structural features of all metal-binding sites, the present analysis also provided an extensive overview of the different behaviors of each metal and their ligand preferences. This information could be of use to experimentalists during the interpretation and fitting of electron-density maps or in the validation of protein structures.

## 2. Methods

MetalPDB (Andreini *et al.*, 2013 ▸; Putignano *et al.*, 2018 ▸) is a database that stores a collection of 3D templates of metal-binding sites. These are automatically extracted from the PDB (Berman *et al.*, 2003 ▸) and describe the local environment around the metal ion(s). Any non-H atom within 3 Å of the metal is identified as one of its donor atoms (DAs), *i.e.* the atoms that directly interact with the metal. The metal ligands are those protein residues or small molecules that contain at least one DA (endogenous or exogenous, respectively). The full metal-binding site contains any other residue or chemical species that has at least one atom within 5.0 Å of a metal ligand. In MetalPDB all atoms of a site have a label according to their structural role (*i.e.* ligand, ligand neighbor, other). This information was exploited during the present analysis to easily access the information of interest.

We retrieved all of the holo sites from the MetalPDB database and used an in-house Python script to compute the metal–DA distances. The script parses each file and differentiates the residues according to the protein chain to which they belong. Only protein DAs were considered in this analysis, and we imposed distance restraints to assign each DA to the correct metal ion in polynuclear sites. Asp and Glu bear a carboxylate group as their side chain (SC). In this work we considered both of their carboxylate O atoms even when only one was labeled as a DA, since the electrostatic charge of the second O atom may still be experienced by the metal. We then plotted all of the computed distances for all of the metal–DA pairs, sub­dividing the data into four resolution ranges: (i) <1.5 Å, (ii) 1.5–2 Å, (iii) 2–2.5 Å and (iv) 2.5–3 Å. A similar approach was used to compute the distances between metals in binuclear sites. Sites containing ligands with partial occupancy were discarded. We did not differentiate the metals according to their oxidation state, as exposure to X-ray radiation may cause the undetected reduction of metal sites (Beitlich *et al.*, 2007 ▸; Strange *et al.*, 2006 ▸; Garman & Weik, 2017 ▸). This phenomenon makes it difficult to ascertain the metal oxidation state on the database scale. To complete our study of the features of metal-binding sites, we retrieved the coordination number for mononuclear sites from MetalPDB. The reported coordination number was extracted by MetalPDB considering all ligands (*i.e.* endogenous, exogenous and water molecules as well). However, the coordination numbers in MetalPDB can underestimate the true values because of unmodeled ligands due to, for example, poor density or partial occupancy. Finally, we computed the number of waters interacting with each metal in mononuclear sites for the <1.5 Å resolution range. Using the SciPy Python module (Virtanen *et al.*, 2020 ▸), we computed the positions of the maximal and integral values for each distance distribution curve. The spread of values in each distribution was quantified by taking the full width at half maximum. All distance distribution statistics are available online in the form of histograms as well as kernel density estimate plots at https://zenodo.org/doi/10.5281/zenodo.10644488.

## 3. Results

We analyzed the coordination preferences and the distribution of metal–DA distances for 115 710 metal-binding sites retrieved from MetalPDB. In the present discussion, we will address each metal individually since it is difficult to generalize the trends for metals belonging to the same group, and it is even more challenging to generalize for all of the inspected metals. All metal ions exhibit markedly diverse behaviors that are closely tied to their electronic configurations and their available oxidation states. The latter also influence the coordination and the composition of the ligands interacting with a given metal. As noted previously (Beitlich *et al.*, 2007 ▸; Strange *et al.*, 2006 ▸; Garman & Weik, 2017 ▸), differentiation of metals based on their oxidation state is not viable due to the potential alterations caused by X-ray irradiation. Indeed, there is no definitive confirmation of the reported oxidation state in the deposited metalloprotein structures. We only analyzed metal–DA pairs with more than 500 entries for each site nuclearity. Unless stated otherwise, we will refer to results obtained for the highest resolution range (<1.5 Å).

**Figure 1:**
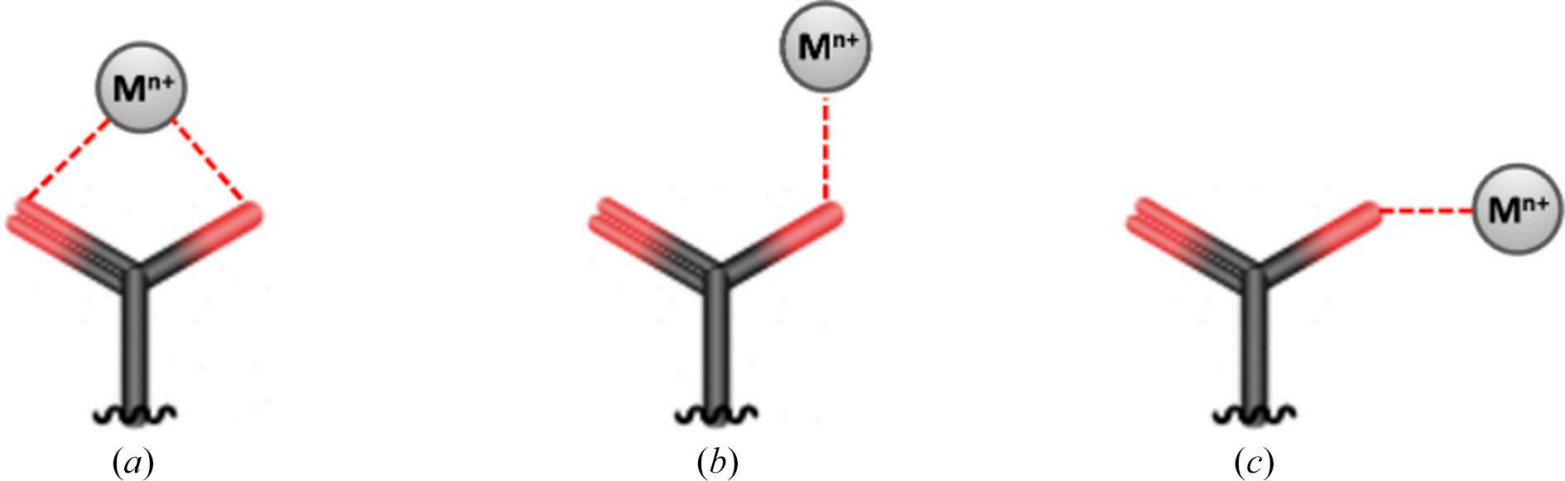
Typical coordinations of the carboxylate group in mononuclear sites: (*a*) bidentate coordination with both O atoms interacting with the metal ion at comparable distances, (*b*) monodentate coordination by OX1, with OX2 in a *syn* orientation with respect to the metal ion, and (*c*) monodentate coordination by OX1, with OX2 in an *anti* orientation. For simplicity, we always label the O atom closer to the metal as OX1.

Based on our results, the carboxylate groups of Asp and Glu are the only functional groups that coordinate almost all of the inspected metals. In Fig. 1 ▸ we report the three typical interactions of this moiety with a metal, which will be discussed in the following sections. Carboxylate coordination is also encountered in polynuclear sites, where this group can act as a connecting bridge between two metal ions (Rardin *et al.*, 1991 ▸).

### 3.1. Alkali and alkali-earth metals

**Figure 2:**
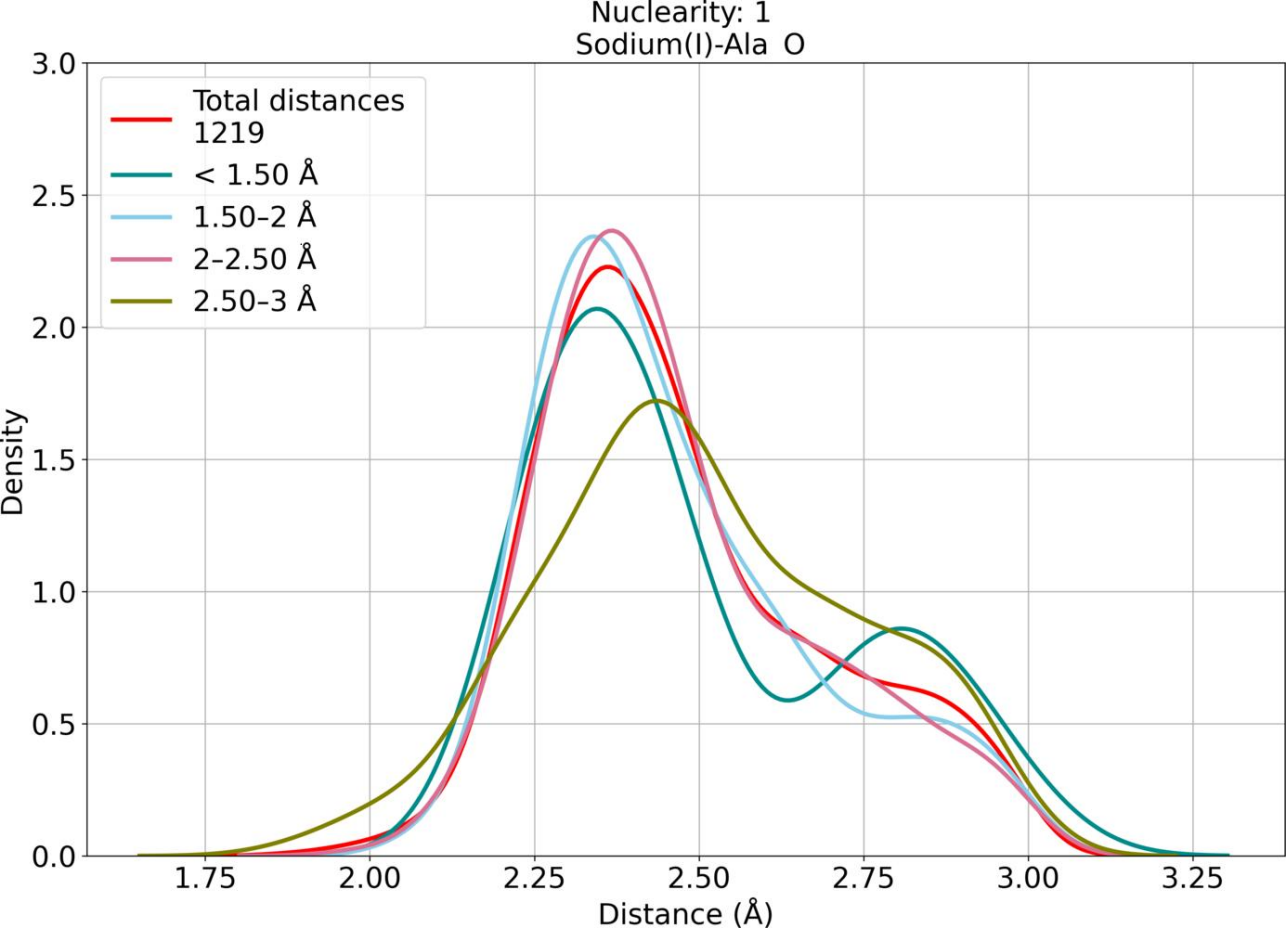
Distance distributions for sodium(I) coordinated by the O atom of Ala. The *y* axis is proportional to the fractional occurrence of the distance in the (sub)data set of interest. The distance statistics are represented as kernel density estimate plots. It is possible that the minor peak at about 2.8 Å may have a relevant contribution from incorrectly modeled water molecules (Nayal & Cera, 1996 ▸; Morshed *et al.*, 2015 ▸; Gohara & Di Cera, 2016 ▸). Histogram representations of the data counts are available online at https://doi.org/10.5281/zenodo.10644488.

**Figure 3:**
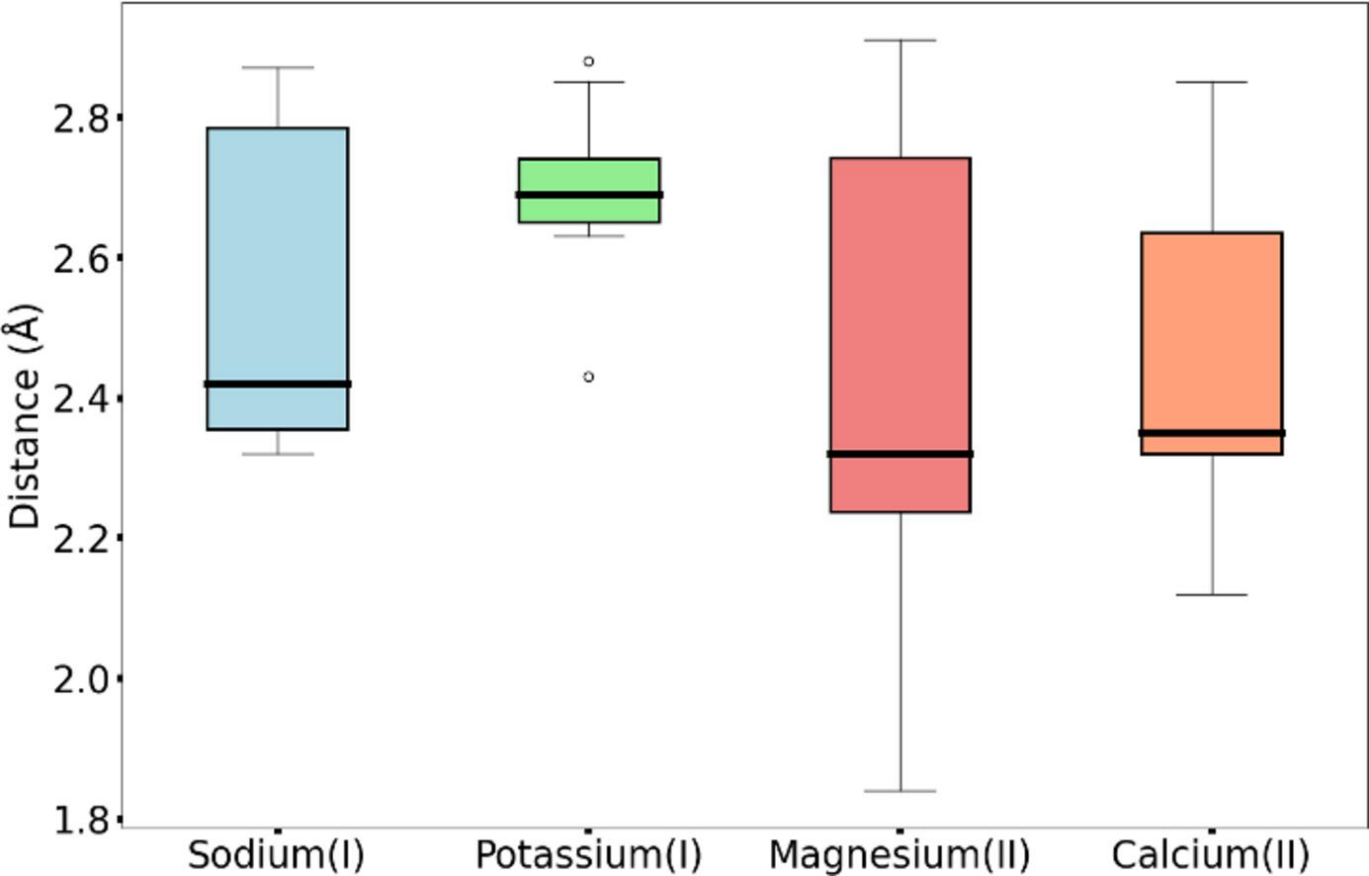
Main-chain O-atom coordination by the alkali and alkaline-earth metals. The plots were computed considering only mononuclear sites in the <1.5 Å resolution range. All of the resulting average distances are detailed in Supplementary Table S2. The parameters of the box plot are as follows: the box range is from the first to the third quartile, the whiskers extend from the 5th to the 95th percentile and the thick line in the box identifies the median. It is possible that the large size of the boxes for sodium(I) and magnesium(II) may include a relevant contribution from incorrectly modeled water molecules (Nayal & Cera, 1996 ▸; Morshed *et al.*, 2015 ▸; Gohara & Di Cera, 2016 ▸).

The typical values for the coordination number of sodium(I) are five (30% of sites) and six (24% of sites) (Supplementary Table S1; Dudev *et al.*, 2018 ▸). Sodium(I) is exclusively coordinated by O atoms of the backbone and side chains (Gln, Asn, Glu and Asp). Despite the DA always being the same, we observe different trends with different ligands. For the backbone O atoms of some residues (Ala, Gln, Ile, Leu, Phe, Ser and Tyr) we observe a major distance peak around 2.35 Å and a minor peak around 2.9 Å (Figs. 2 ▸ and 3 ▸ and Supplementary Table S2), indicating that it is much more common to observe the first distance than the second distance in our data set. An analogous profile appears in the data from the MESPEUS database (Lin *et al.*, 2024 ▸), but the sites corresponding to the second peak in the highest resolution range are flagged as borderline or even dubious by *CMM* (Gucwa *et al.*, 2023 ▸). It is possible that this second peak may have a relevant contribution from incorrectly modeled water molecules (Nayal & Cera, 1996 ▸; Morshed *et al.*, 2015 ▸; Gohara & Di Cera, 2016 ▸); the same problem may lead to an increased width of the distance distributions for sodium(I) coordination (Fig. 3 ▸). Indeed, Arg and Pro produce extremely broad peaks ranging from 2.4 Å to almost 3 Å, whereas for Cys the distribution is slightly less broad (2.2–2.8 Å). Asn, Lys and Val have a single peak at 2.3 Å, with some data skewed towards higher values. The same is observed for Thr coordinating with the main-chain O atom, whereas O^γ1^ gives a peak at 2.8 Å with the highest intensity and a second minor peak at 2.4 Å (Supplementary Table S2). In mononuclear sites, Asp and Glu interact with the metal mainly through the backbone O atom, with a prevalent distance of 2.4 Å. O^δ1^ shows two peaks centered at 2.4 and 2.75 Å, respectively. The distribution of the O^δ2^ distances in mononuclear sites is broad and skewed towards high values (around 4.5 Å), covering all of the possible modes of carboxylate coordination shown in Fig. 1 ▸, although the bidentate configuration is uncommon. In contrast, in binuclear sites we observe two peaks with similar intensity around 3.7 and 4.8 Å (Supplementary Table S3), corresponding to *syn* and *anti* coordination, respectively. For the Glu side chain, O^ɛ1^ shows two peaks with the same intensity around 2.5 and 2.7 Å. The distances of O^ɛ2^ are extremely spread, with increasing densities going from bidentate coordination to monodentate *anti* coordination.

The typical coordination number values for potassium(I) are five (26% of sites), four and six (20% of sites each) (Supplementary Table S1). Potassium(I) can be coordinated by the O atom of the backbone of almost every residue (with no strong evidence for Cys, His and Met; Brás *et al.*, 2014 ▸). For almost every residue type the distances show two different behaviors, either with one peak around 2.7 Å or with a single and very broad peak from 2.6 to 3 Å (Fig. 3 ▸ and Supplementary Table S2). For carboxylate distances we only collected sufficient data for mononuclear sites. O^δ1^ of Asp has the main peak at 2.7 Å, similar to O^ɛ1^ of Glu. O^δ2^ has distances that are evenly distributed from 4 Å to almost 5 Å, corresponding to a preferential monodentate *anti* interaction. O^ɛ2^ of Glu shows extremely spread data, with continuously increasing densities from the bidentate to the monodentate *anti* coordination mode.

**Figure 4:**
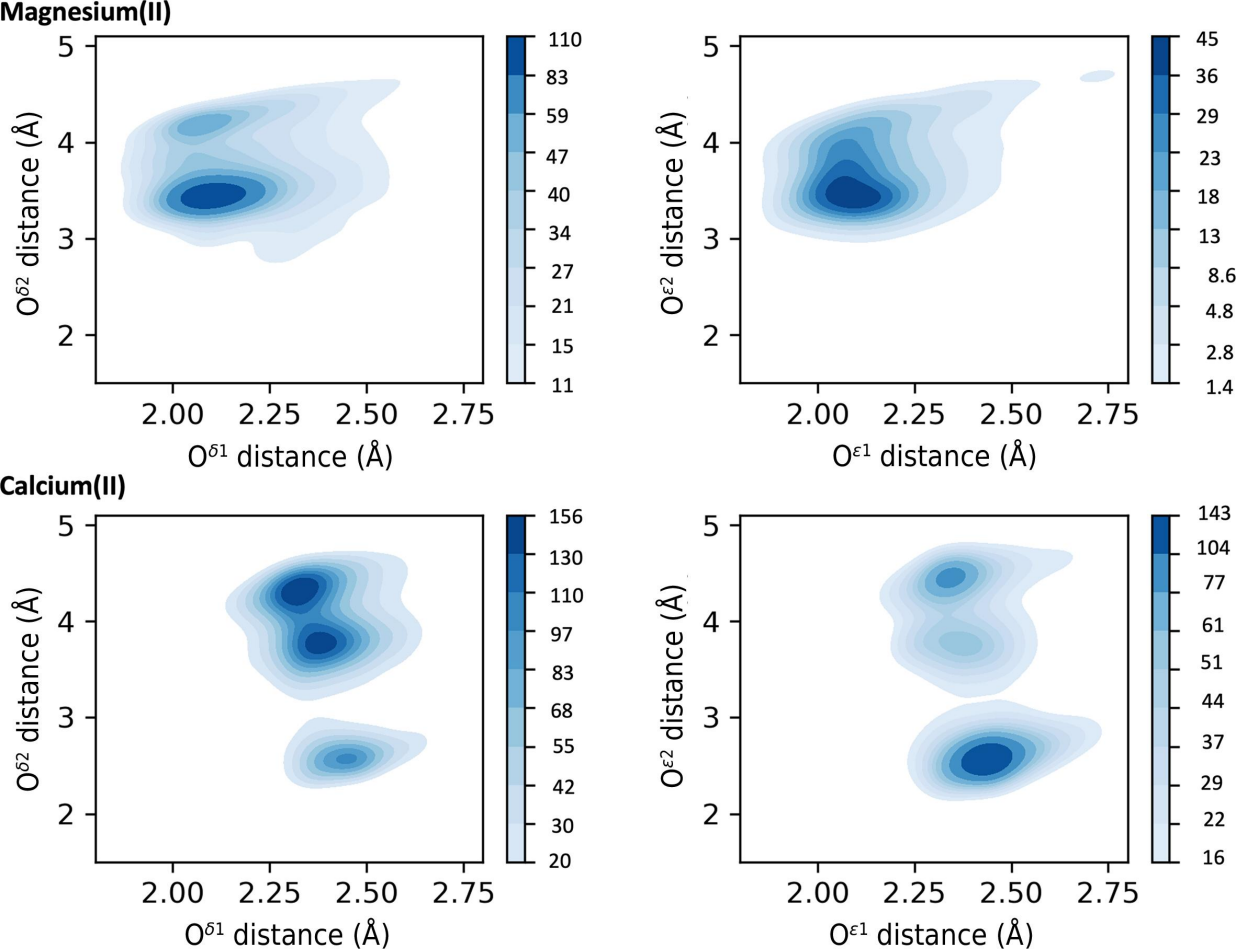
Heatmaps describing the relationship (combined distribution function) involving the distances from the metal ion to O^δ1^/O^δ2^ (left column, Asp) and to O^ɛ1^/O^ɛ2^ (right column, Glu). The heatmaps were plotted using data from all of the analyzed resolution ranges. The top row represents the data for magnesium(II), whereas the bottom row represents the data for calcium(II). The deeper the blue, the more data are present in that region. It can be observed that bidentate coordination is only common for calcium(II), corresponding to the peak at about (2.5, 2.5), especially in the case of Glu coordination (bottom right plot). The scale refers to the number of structures in an area of 0.05 × 0.05 Å.

For magnesium(II) the typical coordination numbers are six (38% of sites) and five (24% of sites), as reported in Supplementary Table S1. An absolute preference for main-chain and SC oxygen coordination is observed, as for the preceding metals. Additionally, His SC coordination is also present. For most residues coordinating using the main-chain O atom (for example Ala, Ile, Val and Asp) there is one major peak (around 2.25 Å) followed by a minor peak (around 2.8 Å), which again may include a contribution from incorrectly modeled water molecules as previously discussed for sodium(I). Less frequently this trend is inverted, such as for Asn. On the other hand, we have some exceptions such as Arg with a single peak at 2.6 Å, and Ser and Leu with two peaks of comparable intensity (Supplementary Table S2). The side-chain O atom of Asn gives a single peak around 2.1 Å, whereas for Gln, Ser and Thr side chains there is an additional peak at 2.8 Å with lower density. His N^δ1^ has a peak around 2.1 Å and a minor peak at about 2.7 Å. Coordination by His N^ɛ2^ is somewhat more common, and the corresponding distance distribution has a single peak at 2.2 Å. Both Asp and Glu feature an OX1 distance of around 2.1 Å, with the data skewed towards higher values. The peaks for OX2 correspond to the *syn* and *anti* monodentate coordinations, with no evidence of bidentate interaction (Fig. 4 ▸). In the same fashion, we tried to analyze the coordination of magnesium(II) by DAs from nucleic acids. A lack of sufficient data, especially in the optimal resolution range (*i.e.* <1.5 Å), prevented the definition of characteristic distances and a thorough investigation.

For calcium(II) we observe a preference for coordination numbers of seven (34% of sites) and six (26% of sites) (Supplementary Table S1). Calcium(II), like the previous metals, has an absolute preference for oxygen coordination. All amino acids other than Met and Cys can participate in calcium(II) coordination through the main-chain O atom, with a distance of around 2.3 Å (Fig. 3 ▸ and Supplementary Table S2). In some cases we observe the occurrence of minor peaks at higher distances caused by the low number of distances available in the considered range. The distance of O^δ1^ of Asp is centered at 2.4 Å, whereas O^ɛ1^ of Glu features two peaks of similar intensity at 2.3 and 2.4 Å. The OX2 distribution has three peaks that correspond to all three of the interaction modes shown in Fig. 1 ▸. The occurrence of the different interaction modes can be appreciated in Fig. 4 ▸, where we plot the distances of OX1 versus OX2, with a comparison with magnesium(II). Curiously, the MESPEUS web interface (Lin *et al.*, 2024 ▸) does not report the occurrence of any bidentate calcium(II) sites, perhaps due to too stringent a threshold being imposed on the similarity between the OX1–metal and OX2–metal distances. Instead, Fig. 4 ▸ shows that bidentate coordination is common for calcium(II), especially with Glu, which is in line with previous observations (Harding, 2006 ▸). Calcium(II) and magnesium(II) are the only metals for which it was possible to gather significant data for carboxylate coordination in sites with a nuclearity of up to three (Supplementary Tables S3 and S4), suggesting a strong preference for interaction with this moiety.

### 3.2. Transition metals

Manganese has coordination numbers of six (47% of sites) and five (22% of sites) (Supplementary Table S1). The main ligands in mononuclear manganese sites are the side chains of His, Asp and Glu. For His, our data confirmed the preference for N^ɛ2^ coordination (3277 distances measured) with a density peak at 2.2 Å, despite the fact that the tautomer with an available lone pair on N^δ1^ is the most abundant (Christianson, 1997 ▸). Carboxylate coordination by Asp and Glu is prevalently in a monodentate *syn* fashion, with a few cases of bidentate and *anti* coordination in mononuclear sites (Supplementary Table S2). Asp also shows the same coordination trends in dinuclear sites (Supplementary Table S3). Glu, on the other hand, prefers *syn* coordination. This is especially clear in dinuclear sites, where most of the O^ɛ2^ distances are centered at 3.4 Å. The above observations are at variance with previous claims that carboxylate–manganese(II) interaction is equally likely to happen with the same preference for *syn* and *anti* stereochemistry, whereas manganese(III) was proposed to prefer *anti* carboxylate coordination (Christianson, 1997 ▸).

**Figure 5:**
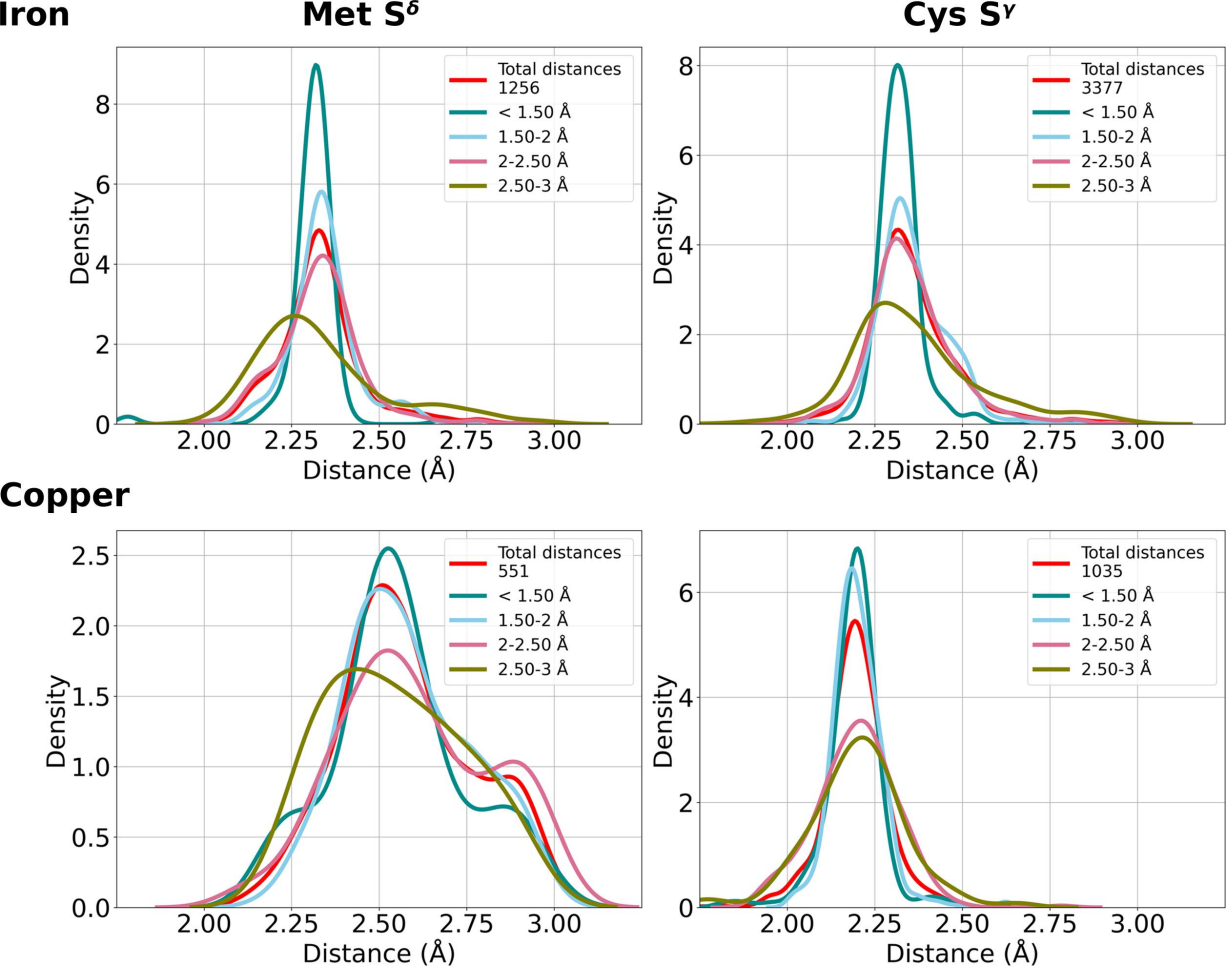
Distance distributions for iron (top row) and copper (bottom row) coordination by the S^δ^ atom of Met (left column) and the S^γ^ atom of Cys (right column). The *y* axis is proportional to the fractional occurrence of the distance in the (sub)data set of interest. The distance statistics are represented as kernel density estimate plots. Histogram representations of the data counts are available online at https://doi.org/10.5281/zenodo.10644488.

For iron the preferred coordination numbers are six (56% of sites) and five (31% of sites) (Supplementary Table S1). Iron is mainly coordinated by the SCs of Met, Cys, Asp, Glu, His and Tyr. Mononuclear sites bearing Met S^δ^ are commonly found in heme proteins (Bertini *et al.*, 2001 ▸) and feature a distance distribution centered at 2.3 Å (Figs. 5 ▸ and 6 ▸ and Supplementary Table S2). His is present in many different types of iron proteins. In our data set it interacts with the iron ion prevalently using the N^ɛ2^ atom; indeed, we detected more than 14 000 instances of N^ɛ2^ coordination in mononuclear sites compared with a few hundred for N^δ1^. Cys shows a sharp peak around 2.3 Å (Figs. 5 ▸ and 6 ▸ and Supplementary Table S2) in all sites regardless of the nuclearity. Tyr is only present in mononuclear sites, with a target distance of 2.0 Å. Glu and Asp carboxylate groups can stabilize high-valent iron intermediates (Bertini *et al.*, 2001 ▸) through their negative charge. For both Asp and Glu (Fig. 7 ▸) in mononuclear sites the OX1 peaks are around 2.1 Å, with data skewed towards higher values. The distance distributions of their OX2 atoms show peaks typical of the bidentate interaction (2.5 Å), as well as in the monodentate *syn* (3.4 Å), which is prevalent, and *anti* (4.2 Å) fashions (Fig. 7 ▸). The distributions are similar in dinuclear sites (Supplementary Table S3). As the above data are related to iron ions in all types of sites, we investigated whether the distributions for iron in heme sites differed. All peak values within the spread of the distribution were the same and only the widths of the distributions varied.

**Figure 6:**
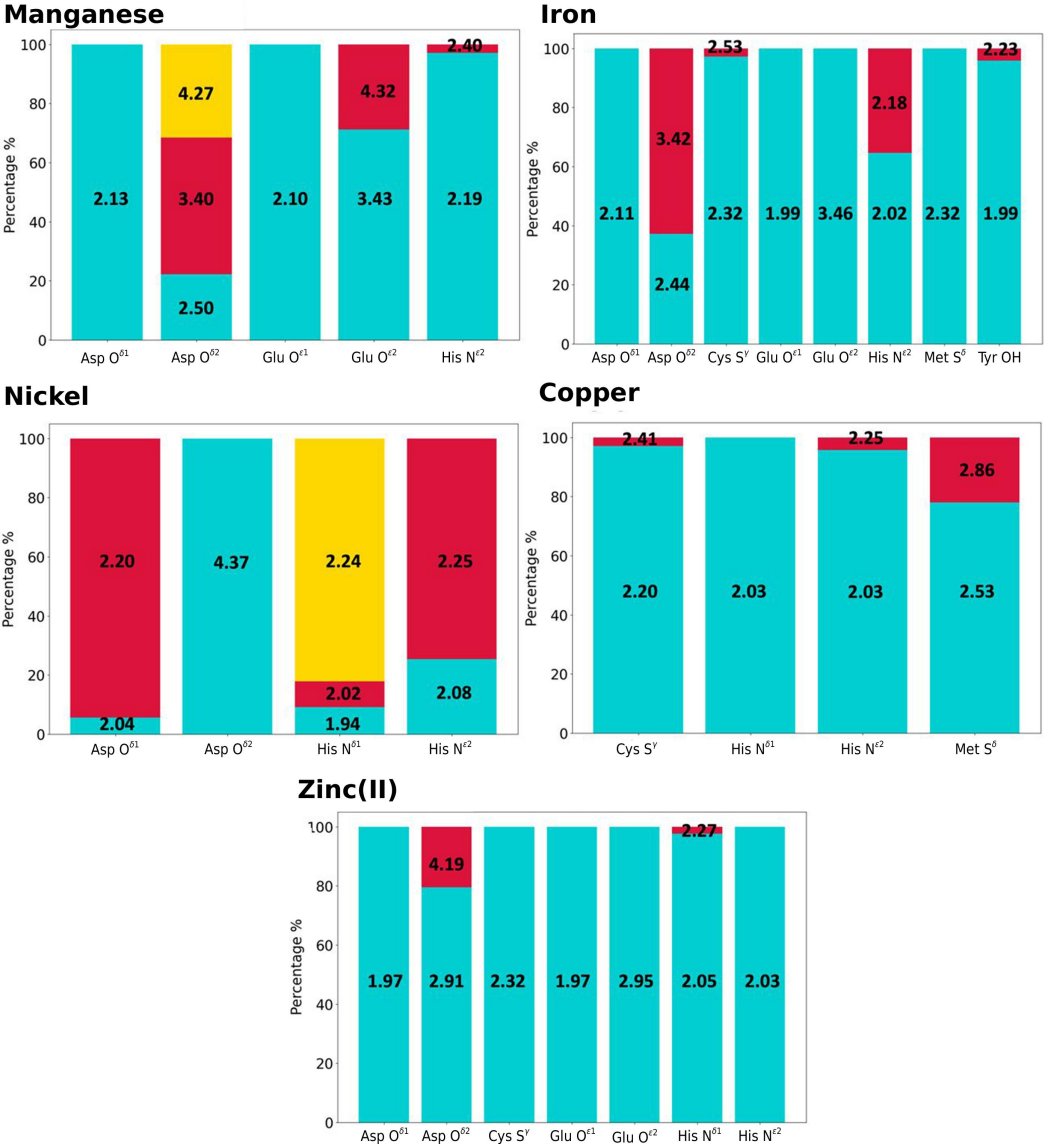
Overview of the distances between transition-metal ions and their donor atoms. For each transition metal–DA pair, the center of the peak(s) of their distance distribution curve was computed (reported in Å in the boxes); only peaks with a density higher than 0.2 were analyzed. Moreover, the integral of each peak is also reported (as a percentage of the total area) to describe the contribution of each peak to the distribution. These values were computed considering only mononuclear sites in the highest range resolution, *i.e.* <1.5 Å. Manganese, iron, nickel and copper can have different oxidation states that are difficult to accurately identify. For this reason we did not report them with their charge as performed for zinc(II). All of the computed values are given in Supplementary Table S2.

**Figure 7:**
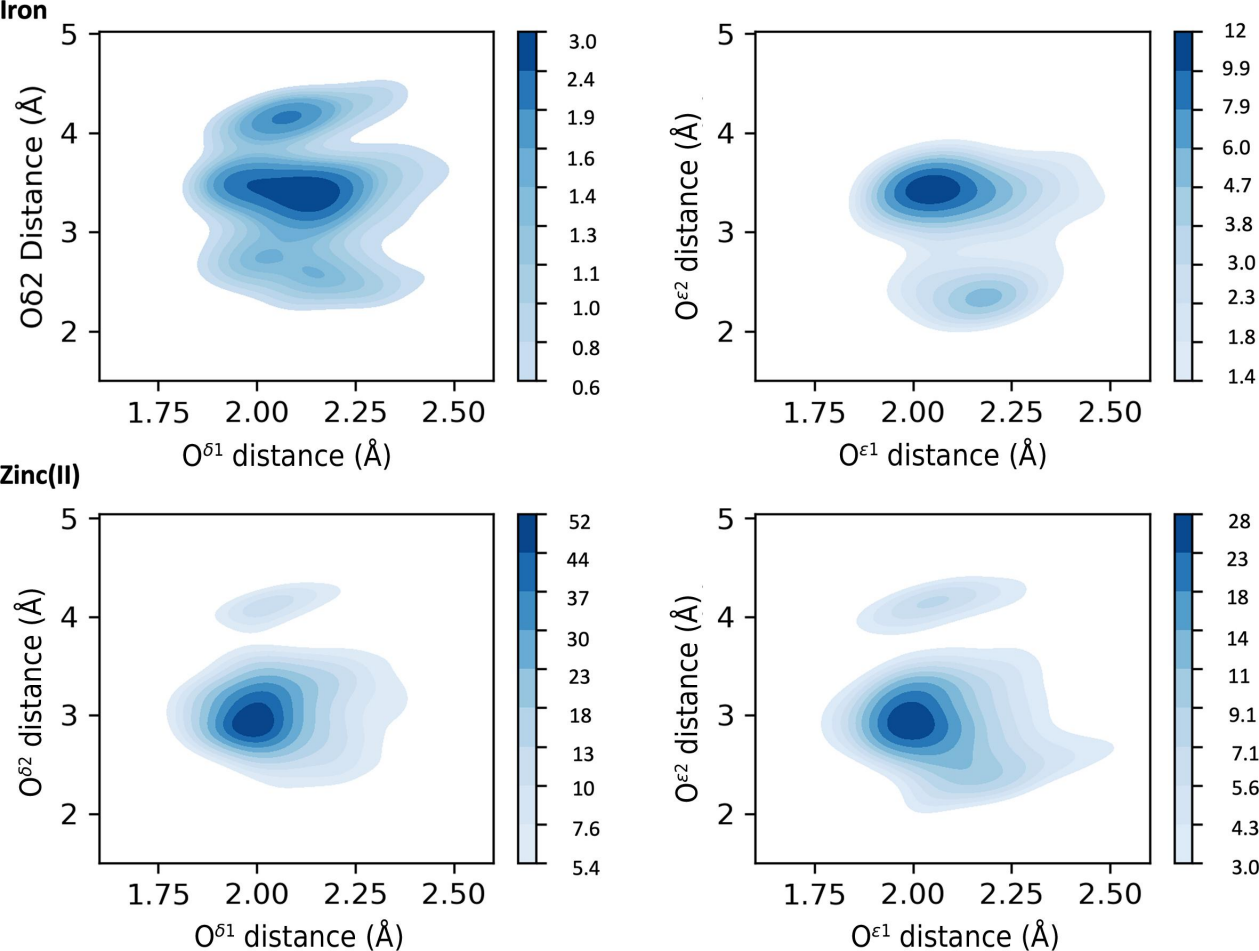
Heatmaps describing the relationship (combined distribution function) involving the distances from the metal ion to O^δ1^/O^δ2^ (left column, Asp) and to O^ɛ1^/O^ɛ2^ (right column, Glu). The heatmaps were plotted using the data from all the resolution ranges analyzed. The top row shows the data for iron, whereas the bottom row shows the data for zinc(II). The deeper the blue, the more data are present in that region. Monodentate *syn* coordination is the most common configuration in all four cases. The scale refers to the number of structures in an area of 0.05 × 0.05 Å.

Coordination numbers of six (41% of sites) and four (17% of sites) are common for nickel (Supplementary Table S1). Its coordination in mononuclear sites is performed by the SCs of His and Asp. Cys and Glu are also known to participate in its coordination (Maroney & Ciurli, 2014 ▸; Maroney, 1999 ▸). For His coordination with the N^δ1^ atom, we observed two nearby density peaks at around 2.0 Å. They correspond to sites where the metal is chelated by the His plus an adjacent residue; the latter is often a Gly coordinating to the backbone N atom, as detected in some SH3 domains (Bacarizo *et al.*, 2014 ▸). The most common nickel–N^δ1^ distance is centered at 2.2 Å (Fig. 6 ▸ and Supplementary Table S2). The different observed values may be linked to different site geometries. The N^ɛ2^ interaction presents a first peak at 2.1 Å and a second peak at 2.2 Å, which aligns with the optimal distance documented for other transition metals (Harding, 2006 ▸). Sufficient data for Asp were only collected for mononuclear sites (Supplementary Table S2); coordination occurs both in the *syn* and *anti* fashions. However, it must be noted that most of the data for the latter are due to the deposition of multiple structures of the same protein in complex with a library of different small organic molecules.

Copper has coordination numbers of four (46% of sites) and three (31% of sites) (Supplementary Table S1). Cys, His and Met SCs are the main ligands of copper-binding sites. The main-chain O and N atoms and the carboxylate groups of Asp and Glu are known copper ligands, but in the present work we did not obtain sufficient data to analyze these interactions. For Cys a peak at 2.20 Å (Figs. 5 ▸ and 6 ▸ and Supplementary Table S2) arises from the formation of a strong covalent bond to the copper ion, with extensive charge transfer from the thiolate to the metal (Olsson & Ryde, 1999 ▸). The coordination of copper by the Met S^δ^ atom is more variable, with a major peak at 2.5 Å (Figs. 5 ▸ and 6 ▸ and Supplementary Table S2). This may be due to the weaker interaction with the Met S atom, which is neutral, whereas the Cys thiolate group bears a net negative charge. His coordination with N^δ1^ and N^ɛ2^ is equally common in mononuclear sites, with the metal–DA distance distributions for both atoms having density peaks at 2.0 Å. In di­nuclear sites, sufficient data were only available for the N^ɛ2^ atom, with the distance distribution peaking at around 2.1 Å (Supplementary Table S3). At a distance of 2.25 Å, N^ɛ2^ has a tail that corresponds to heteronuclear sites where a zinc(II) ion is coordinated by N^δ1^ and the copper ion by N^ɛ2^. The catalytic role of the copper in these sites may explain the larger distance, since the latter can be modulated by changes in the oxidation state (Harding, 2001 ▸). In trinuclear sites we only have data available for N^ɛ2^, with two different peaks of same intensity. From a visual inspection of the sites, we can ascribe this behavior to a typical disposition of this residue in multiple His sites.

Zinc(II) mainly has a coordination number of four (61% of sites; Supplementary Table S1) and its coordination is performed by the SCs of His, Cys, Asp and Glu. The fact that His and Cys can interact in only one possible way (*i.e.* a single DA coordinates the metal ion), together with zinc(II) having a single available oxidation state, can be used to justify the presence of high, sharp peaks in all sites regardless of the nuclearity. For His N^δ1^ we observe some data skewed towards larger distance values even in the highest resolution range. In these cases, the steric hindrance of the protein backbone plays a relevant role and the coordination of the metal ion in catalytic sites must allow transient distortions so that the reactions can take place. The interaction of the zinc(II) ion(s) with the SCs of Asp and Glu is similar in all sites, regardless of the nuclearity (Supplementary Tables S2–S4). While the distance for OX1 is narrow and centered at 1.97 Å (Fig. 6 ▸ and Supplementary Table S2), the distances for OX2 show broad distributions over values typical for bidentate (with lower density) and monodentate *syn* and *anti* coordinations.

The tables of distances computed for lower resolution ranges are available in Supplementary Tables S5–S7.

### 3.3. Metal–metal distances in dinuclear metal sites

**Figure 8:**
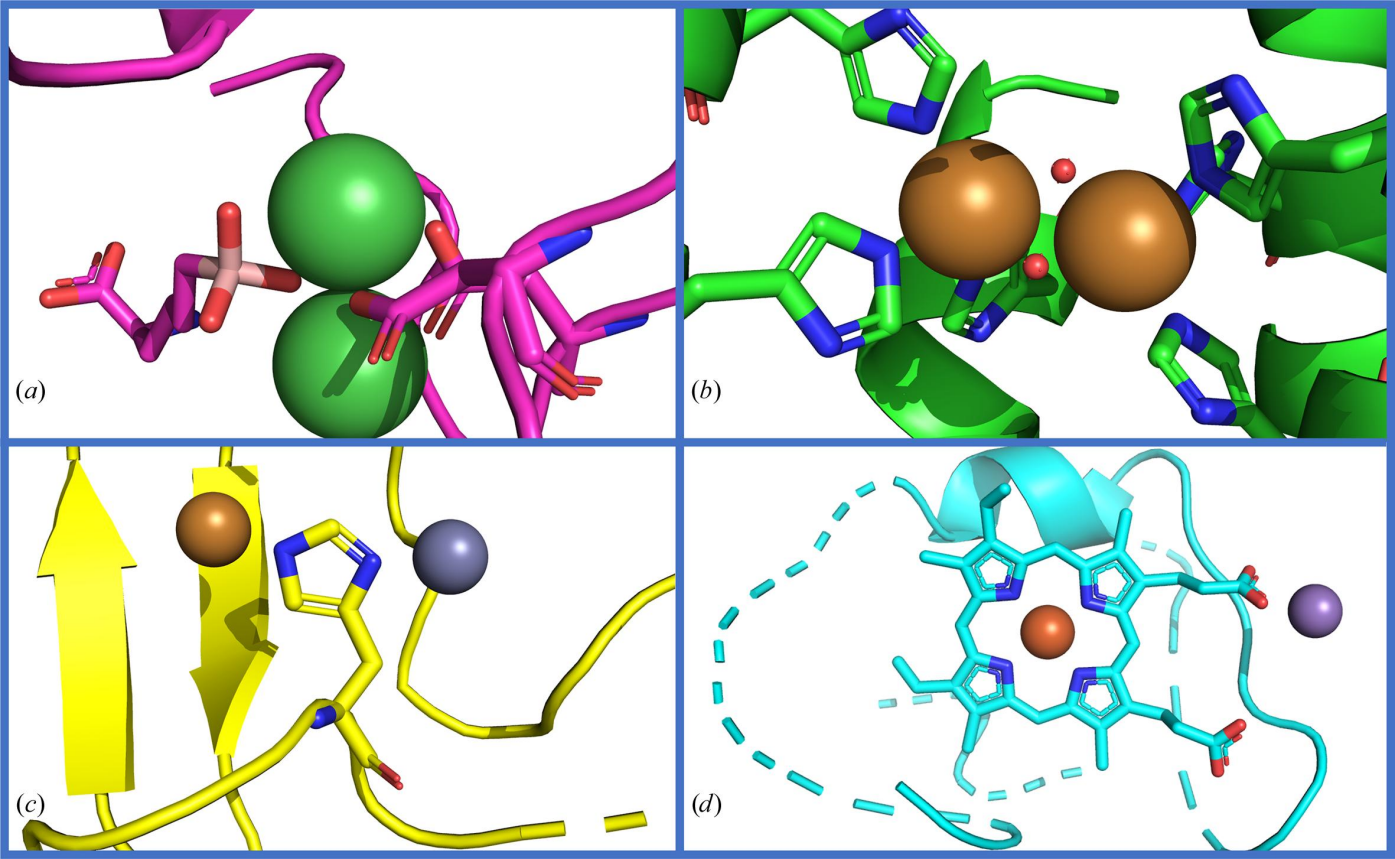
Examples of the different configurations observed in dinuclear sites. (*a*) A homonuclear nickel site with an exogenous molecule and a carboxylate coordinating the two metal ions (PDB entry 4gsv), (*b*) a homonuclear copper site with two bridging water molecules (PDB entry 1ll1), (*c*) zinc(II) and copper ions separated by a histidinate (PDB entry 1sos) and (*d*) a heteronuclear site of iron and manganese within the porphyrin framework (PDB entry 2boq).

For this analysis, multinuclear sites were subdivided into homonuclear and heteronuclear: binding the same or a different metal, respectively. In these two groups, we observe different distributions of inter-metal distances (the peak values and contributions are reported in Supplementary Tables S8–S15), with the latter showing increased density at higher values. In the distance range 3–7 Å the metals can be coordinated by the carboxylate group, with each O atom interacting with a metal or one O atom interacting exclusively with one metal ion and the other acting as a bridge (Fig. 8 ▸ *a*). In addition to water or small anion(s) (for example azide), small organic molecules bridging the two metals with a single DA are also found (Fig. 8 ▸ *b*). As the distances increase the metals can be coordinated by the protein or DNA in different regions without bridging DAs, or by a ligand interacting with each metal with a DA on different molecule branches.

In the distance range 9–11 Å, especially in heteronuclear sites, we observed residues that coordinate one metal with the SC and the second ion with the O atom of the backbone, or a His that coordinates each metal with a different N atom of the side chain (histidinate; Fig. 8 ▸ *c*). For sites with iron these long distances can be due to a heme group that harbors the iron in the porphyrin framework, whereas the second metal is coordinated by the propionate groups (Fig. 8 ▸ *d*).

### 3.4. Metal coordination by water molecules

Although the identification of water molecules bound to metal ions can be challenging, especially when the resolution is less than optimal and is complicated by crystal symmetry operations, with a large data set such as ours it is possible to identify meaningful trends (Supplementary Table S16). Among the alkali and alkaline-earth metals, potassium(I) is the metal for which the lowest occurrence of water molecules in the coordination sphere is observed (36% of sites with no waters and less than 10% of sites with three water molecules or more). Magnesium(II) is the metal with the highest solvation, with 13% of sites having four water molecules or more; surprisingly, 24% of magnesium(II) sites have no water molecules in their coordination sphere. Sodium(I) and calcium(II) have fairly similar hydration patterns, with the majority of sites having two (25% and 34% of sites, respectively) or one (23% and 28% of sites, respectively) water molecules in the coordination sphere, and less than 20% of sites with no waters.

The situation is somewhat different for transition-metal ions. Manganese sites have a specific hydration pattern, with as many as 56% sites with one water molecule, 13.5% of sites with two water molecules, 13.5% with no water molecules and about 4% of sites with four water molecules or more. In the case of nickel, 10% of the sites have one water molecule and 74% have none. Iron, copper and zinc(II) are all relatively similar, with the majority of sites having no water molecules in the coordination sphere of the metal (69–81%) and an additional 14–25% of sites having one water molecule. Copper and zinc(II) feature around 5% of sites with two water molecules, whereas this accounts for only 3% of iron sites. Higher hydration is even less common.

Overall, in 88% of the sites binding transition-metal ions, apart from manganese, there are zero or only one water molecules in the coordination sphere of the metal, whereas this figure is 45% for alkali and alkaline-earth sites. Conversely, 2% of transition-metal sites and 16% of alkali and alkaline-earth sites contain at least four water molecules.

## 4. Discussion

The lack of availability of good restraints for the structure refinement of metal sites, together with the experimental limits of the structure-determination process, cause the distances between any given metal ion and its DAs to be quite variable across the PDB. In this study, we aim to identify the most common distances for each metal–DA pair and to establish reliable distance information relevant for structural biology studies of metalloproteins.

An important issue for the type of analysis that we have performed in this work is that metal ions can be misidentified in deposited PDB structures, in particular in the absence of experiments specifically aimed at the determination of the metal that is present in the final crystal. Microbeam proton-induced X-ray emission (PIXE) is a technique that allows the identification and quantification of metals in protein crystals (Grime & Garman, 2023 ▸). PIXE has successfully been applied to single out and correct errors in deposited metalloprotein structures (Grime *et al.*, 2020 ▸). Other options to investigate which metal is present in MP crystals include X-ray absorption near-edge structure (XANES; Ascone & Strange, 2009 ▸) and extended X-ray absorption fine structure (EXAFS; Hummer & Rompel, 2013 ▸; Arcovito & della Longa, 2012 ▸). Furthermore, *in crystallo* UV–visible (Ronda *et al.*, 2015 ▸), Raman (Stoner-Ma *et al.*, 2011 ▸) or infrared (Hall *et al.*, 2015 ▸) spectroscopy can also provide indications of the oxidation state of the metal ion in the same crystal as used for collection of X-ray diffraction data at a synchrotron facility (Dworkowski *et al.*, 2015 ▸). For metalloenzymes, enzyme-activity assays can also be leveraged (Knape *et al.*, 2015 ▸). X-ray emission spectroscopy (XES) is a further tool that has been used in this context on a range of systems with varying hardware configurations (Emamian *et al.*, 2023 ▸; Kern *et al.*, 2013 ▸). The aforementioned techniques are integrative; namely, they inform on the whole metal content in the region that is probed by the beam. The comparison of anomalous maps calculated from diffraction data collected at different selected wavelengths permits the determination of the number and the three-dimensional localization of various metal ions in a metalloprotein structure (Than *et al.*, 2005 ▸). Spatially resolved anomalous dispersion data are another approach to obtain information for individual binding sites of metals, even allowing the identification of the individual oxidation states in systems containing multiple metal ions of the same type (Spatzal *et al.*, 2016 ▸; Einsle *et al.*, 2007 ▸).

Leveraging combined spectroscopic and crystallographic measurements can also be very useful to assess radiation damage to the MP sample. Radiation damage can occur both at room temperature and at cryotemperatures, with broadly similar effects on the obtained data but a significantly higher progression rate in the former case (Garman & Weik, 2017 ▸). In MPs, site-specific damage is caused by the formation of photoelectrons and free-radical species created by the ionizing effects of X-ray radiation, which may promote the reduction of the metal ions (Pfanzagl *et al.*, 2020 ▸; Ebrahim *et al.*, 2019 ▸; Beitlich *et al.*, 2007 ▸). Another consequence of radiation damage that has relevance here, although not specific for MPs, is the decarboxylation of the side chains of Asp and Glu residues (Weik *et al.*, 2000 ▸). This phenomenon affects the coordination distances measured in this work. The *B*_net_ metric has been used to identify damaged side chains in structures deposited in the PDB that were determined at 100 K (Shelley & Garman, 2022 ▸). Strategies to avoid radiation damage include serial crystallography (Pearson & Mehrabi, 2020 ▸), which entails gathering a number of individual diffraction patterns one after the other, each from a microcrystal or from a small, previously unexposed portion of a larger crystal. Serial crystallography is particularly suited for application at X-ray free-electron lasers (XFELs), where the exceptionally high peak brilliance of femtosecond pulses necessitates constant sample replenishment (Chapman *et al.*, 2011 ▸). This form of data collection, known as serial femtosecond crystallography (Boutet *et al.*, 2012 ▸), allows a diffraction pattern to be collected prior to disintegration of the exposed portion or the crystal. Damage-free structures of copper-containing nitrite reductases were obtained using XFEL radiation and serial femtosecond rotation crystallography (Rose *et al.*, 2021 ▸). This study produced several structures of different intermediates along the catalytic cycle of the enzyme with resolutions between 1.0 and 1.48 Å; in all cases the distances between the two copper ions present in nitrite reductase and their corresponding donor atoms were in agreement with the expected values shown in Supplementary Table S2, within our reported spread. Nevertheless, owing to the very high quality of the experimental data, the authors were able to pinpoint some changes in these distances beyond the reported error. If we exclude the structure with PDB code 6zat, where two alternate positions of the copper(II) ion were detected in the so-called T1Cu site, all distance variations were below 0.2 Å (Rose *et al.*, 2021 ▸).

Unfortunately, the information in the PDB does not provide a consistent overview of the application and outcomes of the aforementioned experimental procedures, which is an obstacle from a data-mining perspective. Even when the same publication reports structures in different oxidation states (Hirano *et al.*, 2015 ▸), the same residue identifier (HEM) has been used for oxidized and reduced *b*-type heme cofactors. Indeed, the PDB does not provide distinct residue identifiers for *b*-type or *c*-type (HEC) heme containing either iron(II) or iron(III). In principle, there is the possibility of using identifiers that properly reflect the oxidation state in the case of single metal ions, for example CU versus CU1 for copper(II) and copper(I), respectively, or FE versus FE2 for iron(III) and iron(II), respectively. However, depositors do not consistently use the appropriate identifiers. For example, in a relatively recent study investigating the use of spatially resolved anomalous dispersion to determine the oxidation states of the two iron ions in the binuclear site of sulerythrin, the authors deposited three different structures at three different total average diffraction-weighted doses, thus also taking radiation damage explicitly into account (Lennartz *et al.*, 2022 ▸). Although they determined one ion in each monomer to be more oxidized than the other, with its reduction occurring only at the highest total dose, according to the identifiers used (FE) the three structures contained four iron(III) ions each. In line with the considerations above, both MetalPDB and MESPEUS do not explicitly report the oxidation state of the metals present in PDB structures. To address this problem, an attractive option would be to use tools to automatically extract spectroscopic information from the scientific literature, especially in the light of the recent success of natural language processing approaches (Swain & Cole, 2016 ▸; Beard *et al.*, 2019 ▸; Zheng *et al.*, 2023 ▸). However, tools that can investigate whether specific measurements have been taken in the context of MP crystallization and structure-determination experiments are not yet routinely available (Hu & Buehler, 2023 ▸; Jinge *et al.*, 2023 ▸). Computational methods can be used to pinpoint possible mistakes in the identification of the metal in a deposited structural model, and also for the detection of incorrectly modeled water molecules (Morshed *et al.*, 2015 ▸; Gucwa *et al.*, 2023 ▸; Echols *et al.*, 2014 ▸). However, additional experimental data are needed to possibly amend such mistakes.

Neutron diffraction does not cause damage to MP crystals (Helliwell, 2020 ▸), thus making control of the oxidation state easier. Rubredoxin, a small protein that harbors a single iron ion coordinated by the S^γ^ atoms of four Cys residues, has been subjected to various structural studies using neutron diffraction (Gardberg *et al.*, 2010 ▸; Cuypers *et al.*, 2013 ▸; Meilleur *et al.*, 2013 ▸), with resolutions from 1.05 to 1.75 Å. The average iron–S^γ^ distance over 11 different structures was 2.29 ± 0.08 Å, which is well aligned with our expected distance of 2.32 ± 0.06 Å (Supplementary Table S2). Oxidation-state dependent changes have been addressed in Cuypers *et al.* (2013 ▸), with all changes in the iron–S^γ^ distances being within 0.1 Å, which is within the experimental uncertainty estimated from the *B* factors (Gurusaran *et al.*, 2014 ▸; Kumar *et al.*, 2015 ▸; see also below). Notably, in addition the authors of the latter study deposited all of their structures always using the FE [*i.e.* iron(III)] identifier, so that one cannot automatically identify the structure of reduced rubredoxin (PDB entry 4ar4).

We analyzed a large number (485 069 distances in 115 710 sites; Supplementary Table S17) of distances for different metal–DA pairs. This is a somewhat larger data set than used in previous related work (Tamames & Ramos, 2011 ▸). The distance distributions show different behaviors both among and within metal groups. Nevertheless, we did observe some general trends that were in agreement with the different natures of the metals. An initial observation is that alkali and alkaline-earth metals are coordinated mainly by the O atoms of the main chain as well as of SCs. We attribute this to the hard nature of these metals (Harding, 2002 ▸; Pearson, 1963 ▸). The relative number of protein ligands versus water molecules in the coordination sphere of sodium(I) is one of the determining factors in the selectivity against lithium(I) in binding sites (Dudev *et al.*, 2018 ▸). Alkali and alkaline-earth metals display distance distributions spread over broad value ranges, which may be due to the predominantly electrostatic character of their interaction, as well as to the difficulty of modeling sodium(I) and magnesium(II) in particular. Notably, long-wavelength beamlines such as beamline I23 at Diamond Light Source allow the use of ions such as potassium(I) and calcium(II) as anomalous scatterers and enable their experimental identification in MP crystals (El Omari *et al.*, 2023 ▸). Despite alkali and alkaline-earth metals sharing a preference for backbone O atoms, not all amino acids are equally likely to coordinate them. For example, we did not find significant data for the interaction of potassium(I) and magnesium(II) with Lys, Phe, Pro and Tyr. Moreover, in contrast to all other alkali and alkaline-earth metals, magnesium(II) can also be coordinated by His N^ɛ2^ and N^δ1^.

In contrast to alkali and alkaline-earth metals, the transition metals are seldom bound to main-chain O atoms. Their distance distributions feature narrow peaks centered at a value that is mainly dependent on the chemical nature of the DA. Iron and zinc(II) have the same average distance from Cys S^γ^ (2.32 Å), which is 0.12 Å longer (Figs. 5 ▸, 6 ▸ and Supplementary Table S2) than in the case of copper. Copper and iron are the only two metals coordinated by both the Cys thiolate and the Met S^δ^ atom. For Met coordination the iron–S^δ^ distance is the same as for Cys S^γ^, whereas for copper the distribution of the metal–S^δ^ distance is broader than that of the S^γ^–metal distance, with a higher peak value of 2.5 Å versus 2.3 Å (Figs. 5 ▸, 6 ▸ and Supplementary Table S2). It is known that geometrical factors and site organization can significantly impact the interaction between the Met S^δ^ atom and the copper ion (Olsson & Ryde, 1999 ▸). Indeed, the larger S^δ^–copper distance has been ascribed to the protein chain forcing a longer interaction in cupredoxins, which are the most common family of copper proteins in the PDB. This is reflected in the different amount of charge transferred by the negatively charged thiolate and the neutral Met S atom (Holm *et al.*, 1996 ▸).

For His we observe an overall general preference for N^ɛ2^ coordination, which becomes exclusive for manganese and iron. The metal–N^ɛ2^ distance is similar for magnesium(II), manganese and nickel at around 2.2 Å, whereas it is shorter for iron, copper and zinc(II) at around 2.0 Å. Our data indicate that metal coordination by the N^δ1^ atom of His is significantly less common than by the N^ɛ2^ atom for all metals, which is probably due to steric hindrance of the protein backbone. Notably, the metal–N^δ1^ and metal–N^ɛ2^ distances are typically the same within the spread of the corresponding distributions.

Asp and Glu carboxylate coordination shows the most diverse results for all metals. Sodium(I) coordination by Asp and Glu is typically monodentate, with the *syn* and *anti* configurations (Fig. 1 ▸) being equally common; bidentate interactions are also possible. Glu coordination of potassium(I) resembles that of sodium(I), whereas for Asp the *anti* configuration is prevalent. Magnesium(II) is coordinated almost exclusively in a monodentate way; for this metal, Asp coordinates similarly to sodium(I), whereas Glu is only observed in the *syn* configuration. Calcium(II) is the only metal in the first two groups for which carboxylate coordination can occur in all three possible configurations with comparable frequency. Magnesium(II) and calcium(II) are chemically similar and the difference in their interaction with carboxylate may be due to the larger number of coordinating waters (Supplementary Table S16) present in magnesium(II) sites, which forces the monodentate configuration (Dudev & Lim, 2004 ▸). This behavior is functionally relevant, as it helps enzymes to distinguish between the two metals (Dudev & Lim, 2007 ▸). The interaction of Asp with manganese as well as iron can occur in all three of the possible configurations in Fig. 1 ▸, whereas the binding mode of Glu is mainly *syn*. Nickel only interacts with Asp, and exclusively in an *anti* fashion. For zinc(II) the carboxylate coordination is prevalently monodentate *syn* for both Asp and Glu. Cumulatively, these results indicate that the carboxylate groups of Asp and Glu have metal-dependent configuration preferences. This selectivity is presumably also driven by the volume available within the metal-binding site, which contributes by defining the specific rotameric state of the aliphatic SCs. Intrinsically, this modulates the physiological function of the metal site. For example, in iron sites the carboxylate shift plays an important role in controlling the availability of coordinating positions in the active sites, especially of those with multiple iron ions (Bertini *et al.*, 2001 ▸). In mononuclear sites, the change in carboxylate coordination mode can be a mechanism to control the coordination number of the iron and plays a crucial role in controlling the O_2_ reactivity (Bertini *et al.*, 2001 ▸). Similarly, the carboxylate shift in zinc(II) enzymes is instrumental in determining the substrate-exchange rate (Sousa *et al.*, 2007 ▸).

It is relevant to compare our data, which are based only on experimental protein structures, with previous similar analyses. First of all, we note that our statistics are quite similar to those that can be extracted from the MESPEUS database (Lin *et al.*, 2024 ▸; Hsin *et al.*, 2008 ▸), which is expected given that the underlying structural data are essentially the same. In addition, the present work provides fits for all metal–donor atom distributions, quantifying the central distance values and the spread of the distance density distributions. Our analysis of carboxylate coordination modes is also not available from MESPEUS or from the current web interface of MetalPDB. Harding and coworkers have provided numerous investigations into expected metal–donor atom distances in metalloproteins (Harding, 2006 ▸; Harding *et al.*, 2010 ▸), with smaller data sets than we used here. Our larger data set allowed us to define expected values for each amino acid individually (reported in the supporting information) and, in addition, for less common side-chain atoms such as Asn and Gln. Within the determined spread of values, we did not find significant discrepancies between the results of Harding *et al.* (2010 ▸) and our results. For example, the data for the coordination of alkali and alkaline-earth metals by the backbone carbonyl O atom, as also studied in Harding (2002 ▸) and Gohara & Di Cera (2016 ▸), agree within 0.05 Å, with the corresponding spreads of values being between 0.10 and 0.20 Å. Another useful reference is provided by the approximate error on the distances. Even though PDB structures do not provide error estimates on the atom coordinates and *B* factors (Cruickshank, 1999 ▸), it is possible to approximate the uncertainty of the position of each individual atom by taking into account its own *B* factor versus the average for all atoms (Gurusaran *et al.*, 2014 ▸; Kumar *et al.*, 2015 ▸). Such uncertainties can be propagated to the calculation of errors on the measured distances (Gurusaran *et al.*, 2014 ▸). In our data set, typical errors range from a few hundredths of an ångström in the analyzed structures with the highest resolution up to the order of 1 Å in sites within structures at about 3 Å resolution. Thus, the deviation of 0.05 Å between the different studies mentioned above is largely within experimental error.

In this work, we analyzed metal–donor atom distances regardless of the coordination environment, *i.e.* without taking into account which ligands are included in the metal-binding site. Therefore, we provide a single expected value for a metal paired with a given donor atom in a given amino acid. Quantum-mechanic/molecular-mechanic studies have addressed this aspect. For example, in Li *et al.* (2010 ▸) a structural zinc(II) site was compared with a catalytic zinc(II) site with different zinc(II) ligands, with Cys in common. For the former site, the Zn–S^γ^ distance was in the range 2.29–2.43 Å, with respect to values of 2.20–2.38 Å in the latter site. In a more extensive study, average bond lengths were computed for a number of zinc(II) systems with different ligand configurations (Tamames *et al.*, 2007 ▸). The interaction that experienced the largest span of distances was with the N atom of the side chain of His (not separating N^δ1^ from N^ɛ2^), ranging from 2.03 to 2.17 Å, depending on the ligands present in the site. From our data set, we obtained typical distance values of 2.05 ± 0.06 and 2.03 ± 0.07 Å for N^δ1^ and N^ɛ2^, respectively, whereas Harding reported a value of 2.04 ± 0.04 Å (Harding, 2006 ▸). We can thus conclude that in a few specific cases the effect of the ligand composition in the metal-binding site can result in a small deviation from the expected values reported here and in the previous literature. This is presumably more likely for sites with uncommon ligand compositions, which would constitute only a minor fraction of the PDB contents.

By separating the data set into different resolution ranges, it becomes possible to appreciate how the data distributions change for all metals as the resolution decreases. In the highest range the distributions usually show well defined peaks, whereas as we move to lower resolutions the data become more and more spread and shift towards higher values (compare the values in Supplementary Table S2 with the values in Supplementary Table S5–S7). This is because in high-resolution structures the electron-density maps are more accurate and permit a better determination of the position of the atoms, including the metal ion and the ligands. This decreased uncertainty results in more reliable and well defined metal–ligand distances. For this reason, we only used measurements in the highest resolution range available to compute the mean distances shown in Supplementary Table S2. Such distances can be used as a reference for the determination, refinement and/or validation of novel 3D metalloprotein structures. In addition, the identified metal-specific preferences for different configurations of Asp versus Glu binding can be taken into account not only during the structure-determination/validation process but also in the development of improved force fields for molecular-dynamics simulations (Macchiagodena *et al.*, 2020 ▸; Bazayeva *et al.*, 2023 ▸; Melse *et al.*, 2023 ▸). We also investigated whether our results could be affected by the evolution of structure-determination methods over time by partitioning the whole data set into ten-year blocks based on the date of structure deposition. In practice, we did not observe specific effects, with the exception of the distance distributions becoming narrower in most recent structures and minor changes in the relative importance of the different peaks in distance distributions (data not shown).

## 5. Conclusions

In the present analysis, we have described the coordination behavior and interaction preferences of the metals normally found in proteins. In addition, we have computed distance distributions, and the corresponding maxima, for four resolution ranges. Our data confirm the dependence of the distances reported in currently available structures on the resolution. Using only data derived from structures with a resolution better than 1.5 Å, we derived reference values that can be used by the scientific community for the refinement and validation of structure-determination experiments. Owing to the different widths of the observed distance distributions, we suggest that such target distances should be imposed more loosely for alkali and alkaline-earth metals than for transition metals.

## Supplementary Material

Supplementary Tables. DOI: 10.1107/S2059798324003152/gm5103sup1.pdf

All distance distribution statistics in the form of histograms as well as of kernel density estimate plots.: https://doi.org/10.5281/zenodo.10644488
