# Supplementary material for: A database overview of metal-coordination distances in metalloproteins
Source: Acta Crystallogr D Struct Biol. 2024 Apr 29;80(Pt 5):362–76. doi: 10.1107/S2059798324003152 (PMC11066882; doi:10.1107/S2059798324003152)
Supplement: Supplementary file 1 [file d-80-00362-sup1.pdf]

Acta Cryst D STRUCTURAL BIOLOGY

Volume 80 (2024)

Supporting information for article:

A database overview of metal-coordination distances in metalloproteins

Milana Bazayeva, Claudia Andreini and Antonio Rosato

Table S1 Coordination number (CN) and their relative percentage for all the inspected metals. The results were computed considering a total resolution range of 1-3 Å for the mononuclear sites.

Metal	CN	Number of sites	Percentage
Sodium(I)	1	783	6.5%
Sodium(I)	2	1134	9.4%
Sodium(I)	3	1658	13.8%
Sodium(I)	4	1820	15.1%
Sodium(I)	5	3591	29.8%
Sodium(I)	6	2907	24.1%
Sodium(I)	7	146	1.2%
Sodium(I)	8	7	0.6%
Potassium(I)	1	208	5.9%
Potassium(I)	2	336	9.5%
Potassium(I)	3	530	15.0%
Potassium(I)	4	709	20.1%
Potassium(I)	5	916	26.0%
Potassium(I)	6	709	20.1%
Potassium(I)	7	95	2.7%
Potassium(I)	8	19	0.5%
Magnesium(II)	1	888	4.4%
Magnesium(II)	2	1536	7.6%
Magnesium(II)	3	2260	11.2%
Magnesium(II)	4	2282	11.3%
Magnesium(II)	5	5007	24.7%
Magnesium(II)	6	7742	38.2%
Magnesium(II)	7	499	2.5%

Magnesium(II)	8	28	0.1%
Calcium(II)	1	451	2.4%
Calcium(II)	2	724	3.9%
Calcium(II)	3	1024	5.5%
Calcium(II)	4	1532	8.3%
Calcium(II)	5	2163	11.7%
Calcium(II)	6	4827	26.071%
Calcium(II)	7	6651	35.9%
Calcium(II)	8	1143	6.2%
Manganese	1	114	3.4%
Manganese	2	143	4.2%
Manganese	3	243	7.2%
Manganese	4	357	10.6%
Manganese	5	750	22.3%
Manganese	6	1588	47.2%
Manganese	7	162	4.8%
Manganese	8	9	0.3%
Iron	1	19	0.1%
Iron	2	83	0.6%
Iron	3	178	1.3%
Iron	4	486	3.6%
Iron	5	4173	30.8%
Iron	6	7606	56.2%
Iron	7	960	7.1%
Iron	8	30	0.2%
Nickel	1	99	5.1%
Nickel	2	177	9.1%

Nickel	3	194	10.0%
Nickel	4	336	17.3%
Nickel	5	285	14.7%
Nickel	6	796	41.1%
Nickel	7	46	2.3%
Nickel	8	4	0.2%
Copper	1	61	2.7%
Copper	2	89	3.9%
Copper	3	705	31.3%
Copper	4	1041	46.1%
Copper	5	238	10.6%
Copper	6	114	5.1%
Copper	7	7	0.3%
Copper	8	1	0.04%
Zinc(II)	1	519	2.4%
Zinc(II)	2	1128	5.2%
Zinc(II)	3	1827	8.5%
Zinc(II)	4	13069	60.8%
Zinc(II)	5	3110	14.5%
Zinc(II)	6	1598	7.4%
Zinc(II)	7	244	1.1%
Zinc(II)	8	9	0.4%

Table S2 Peak(s) center and the contribution to the distributions computed for each metal-donor atom (DA) pair in mononuclear sites. These values were computed for the < 1.5 Å resolution range for peaks with a density higher than 0.2.

**Mononuclear sites, resolution range < 1.5 Å**					
**Metal**	**Residue**	**DA**	**Peak (Å)**	**Std (Å)**	**Peak contribution**
Sodium(I)	Ala	O	2.4	0.2	72.5%
Sodium(I)	Ala	O	2.8	0.2	27.5%
Sodium(I)	Arg	O	2.4	0.2	85.8%
Sodium(I)	Arg	O	2.8	0.2	14.2%
Sodium(I)	Asn	O	2.3	0.2	100.0%
Sodium(I)	Asn	Oδ1	2.4	0.2	79.1%
Sodium(I)	Asn	Oδ1	2.8	0.2	20.9%
Sodium(I)	Asp	O	2.4	0.2	73.9%
Sodium(I)	Asp	O	2.7	0.2	26.1%
Sodium(I)	Asp	Oδ1	2.4	0.2	69.3%
Sodium(I)	Asp	Oδ1	2.8	0.2	30.7%
Sodium(I)	Asp	Oδ2	3.7	0.4	54.0%
Sodium(I)	Asp	Oδ2	4.3	0.4	46.0%
Sodium(I)	Cys	O	2.4	0.1	100.0%
Sodium(I)	Gln	O	2.4	0.2	50.8%
Sodium(I)	Gln	O	2.8	0.2	49.2%
Sodium(I)	Gln	Oε1	2.4	0.3	56.3%
Sodium(I)	Gln	Oε1	2.8	0.3	43.8%
Sodium(I)	Glu	O	2.4	0.2	100.0%
Sodium(I)	Glu	Oε1	2.4	0.3	60.6%
Sodium(I)	Glu	Oε1	2.7	0.3	39.5%
Sodium(I)	Glu	Oε2	3.6	0.5	52.5%

Sodium(I)	Glu	Oε2	4.4	0.5	47.5%
Sodium(I)	Gly	O	2.3	0.2	64.2%
Sodium(I)	Gly	O	2.8	0.2	35.9%
Sodium(I)	Ile	O	2.4	0.2	71.1%
Sodium(I)	Ile	O	2.8	0.2	28.9%
Sodium(I)	Leu	O	2.3	0.3	58.2%
Sodium(I)	Leu	O	2.8	0.3	41.8%
Sodium(I)	Lys	O	2.4	0.2	87.1%
Sodium(I)	Lys	O	2.7	0.2	6.7%
Sodium(I)	Lys	O	2.8	0.2	6.2%
Sodium(I)	Phe	O	2.3	0.2	68.2%
Sodium(I)	Phe	O	2.7	0.2	31.8%
Sodium(I)	Pro	O	2.5	0.3	48.6%
Sodium(I)	Pro	O	2.8	0.2	51.5%
Sodium(I)	Ser	O	2.3	0.2	74.8%
Sodium(I)	Ser	O	2.9	0.2	25.2%
Sodium(I)	Ser	Oγ	2.5	0.2	68.1%
Sodium(I)	Ser	Oγ	2.9	0.2	31.9%
Sodium(I)	Thr	O	2.4	0.2	100.0%
Sodium(I)	Thr	Oγ1	2.4	0.2	26.8%
Sodium(I)	Thr	Oγ1	2.8	0.2	73.2%
Sodium(I)	Tyr	O	2.4	0.2	73.3%
Sodium(I)	Tyr	O	2.8	0.2	26.7%
Sodium(I)	Val	O	2.3	0.2	84.8%
Sodium(I)	Val	O	2.8	0.2	15.2%
Potassium(I)	Ala	O	2.9	0.2	100.0%
Potassium(I)	Asn	Oδ1	2.3	0.2	6.0%

Potassium(I)	Asn	Oδ1	2.7	0.2	36.2%
Potassium(I)	Asn	Oδ1	2.9	0.2	57.9%
Potassium(I)	Asp	O	2.7	0.1	100.0%
Potassium(I)	Asp	Oδ1	2.8	0.2	100.0%
Potassium(I)	Asp	Oδ2	4.2	0.4	100.0%
Potassium(I)	Glu	Oε1	2.0	0.3	34.4%
Potassium(I)	Glu	Oε1	2.6	0.3	65.6%
Potassium(I)	Glu	Oε2	3.0	0.3	69.2%
Potassium(I)	Glu	Oε2	4.4	0.5	30.8%
Potassium(I)	Gly	O	2.4	0.1	8.1%
Potassium(I)	Gly	O	2.7	0.1	91.9%
Potassium(I)	Leu	O	2.7	0.1	100.0%
Potassium(I)	Ser	O	2.7	0.2	100.0%
Potassium(I)	Ser	Oγ	2.5	0.1	9.5%
Potassium(I)	Ser	Oγ	2.9	0.1	90.5%
Potassium(I)	Thr	O	2.6	0.1	27.3%
Potassium(I)	Thr	O	2.9	0.1	72.7%
Potassium(I)	Val	O	2.7	0.1	100.0%
Magnesium(II)	Ala	O	2.3	0.2	78.6%
Magnesium(II)	Ala	O	2.8	0.2	21.4%
Magnesium(II)	Arg	O	2.6	0.2	100.0%
Magnesium(II)	Asn	O	2.3	0.2	11.4%
Magnesium(II)	Asn	O	2.7	0.2	88.6%
Magnesium(II)	Asn	Oδ1	2.1	0.2	100.0%
Magnesium(II)	Asp	O	2.1	0.3	86.3%
Magnesium(II)	Asp	O	2.9	0.3	13.7%
Magnesium(II)	Asp	Oδ1	2.0	0.2	100.0%

Magnesium(II)	Asp	Oδ2	3.4	0.4	70.5%
Magnesium(II)	Asp	Oδ2	4.1	0.4	29.5%
Magnesium(II)	Gln	Oε1	2.3	0.2	88.4%
Magnesium(II)	Gln	Oε1	2.8	0.2	11.6%
Magnesium(II)	Glu	Oε1	2.1	0.2	85.6%
Magnesium(II)	Glu	Oε1	2.4	0.2	14.4%
Magnesium(II)	Glu	Oε2	3.4	0.3	100.0%
Magnesium(II)	Gly	O	2.3	0.2	85.7%
Magnesium(II)	Gly	O	2.8	0.2	14.3%
Magnesium(II)	His	Nδ1	2.1	0.2	90.4%
Magnesium(II)	His	Nδ1	2.8	0.2	9.6%
Magnesium(II)	His	Nε2	2.2	0.1	100.0%
Magnesium(II)	Ile	O	1.2	0.2	6.7%
Magnesium(II)	Ile	O	2.3	0.2	93.3%
Magnesium(II)	Leu	O	2.1	0.3	39.8%
Magnesium(II)	Leu	O	2.8	0.3	60.2%
Magnesium(II)	Ser	O	2.2	0.3	60.8%
Magnesium(II)	Ser	O	2.7	0.3	39.2%
Magnesium(II)	Ser	Oγ	2.1	0.2	92.9%
Magnesium(II)	Ser	Oγ	2.9	0.2	7.1%
Magnesium(II)	Thr	O	2.2	0.3	100.0%
Magnesium(II)	Thr	Oγ1	2.1	0.2	91.2%
Magnesium(II)	Thr	Oγ1	2.7	0.2	8.8%
Magnesium(II)	Val	O	2.3	0.2	92.9%
Magnesium(II)	Val	O	2.9	0.2	7.1%
Calcium(II)	Ala	O	2.3	0.1	89.2%
Calcium(II)	Ala	O	2.8	0.1	10.8%

Calcium(II)	Arg	O	2.1	0.1	15.3%
Calcium(II)	Arg	O	2.4	0.1	84.7%
Calcium(II)	Asn	O	2.3	0.1	100.0%
Calcium(II)	Asn	Oδ1	2.4	0.1	100.0%
Calcium(II)	Asp	O	2.4	0.1	100.0%
Calcium(II)	Asp	Oδ1	2.4	0.1	100.0%
Calcium(II)	Asp	Oδ2	2.6	0.3	22.5%
Calcium(II)	Asp	Oδ2	3.8	0.4	43.1%
Calcium(II)	Asp	Oδ2	4.2	0.3	34.3%
Calcium(II)	Gln	O	2.3	0.1	100.0%
Calcium(II)	Gln	Oε1	2.3	0.1	94.2%
Calcium(II)	Gln	Oε1	2.6	0.1	5.8%
Calcium(II)	Glu	O	2.3	0.1	93.7%
Calcium(II)	Glu	O	2.6	0.1	3.2%
Calcium(II)	Glu	O	2.8	0.1	3.1%
Calcium(II)	Glu	Oε1	2.3	0.1	52.5%
Calcium(II)	Glu	Oε1	2.4	0.1	47.5%
Calcium(II)	Glu	Oε2	2.6	0.3	32.2%
Calcium(II)	Glu	Oε2	3.7	0.5	30.0%
Calcium(II)	Glu	Oε2	4.5	0.4	37.8%
Calcium(II)	Gly	O	2.3	0.1	100.0%
Calcium(II)	His	O	2.40	0.09	92.3%
Calcium(II)	His	O	2.6	0.1	7.7%
Calcium(II)	Ile	O	2.3	0.1	100.0%
Calcium(II)	Leu	O	2.3	0.2	86.3%
Calcium(II)	Leu	O	2.7	0.2	13.8%
Calcium(II)	Lys	O	2.32	0.08	93.5%

Calcium(II)	Lys	O	2.64	0.08	6.6%
Calcium(II)	Phe	O	2.3	0.1	100.0%
Calcium(II)	Pro	O	2.4	0.1	95.7%
Calcium(II)	Pro	O	2.8	0.1	4.3%
Calcium(II)	Ser	O	2.3	0.1	93.5%
Calcium(II)	Ser	O	2.7	0.1	6.5%
Calcium(II)	Ser	Oγ	2.5	0.1	93.0%
Calcium(II)	Ser	Oγ	3.0	0.1	7.0%
Calcium(II)	Thr	O	2.4	0.1	92.0%
Calcium(II)	Thr	O	2.9	0.1	8.3%
Calcium(II)	Thr	Oγ1	2.5	0.1	94.6%
Calcium(II)	Thr	Oγ1	2.7	0.1	5.4%
Calcium(II)	Tyr	O	2.36	0.07	100.0%
Calcium(II)	Val	O	2.3	0.1	100.0%
Manganese	Asp	Oδ1	2.1	0.1	100.0%
Manganese	Asp	Oδ2	2.5	0.5	22.3%
Manganese	Asp	Oδ2	3.4	0.6	46.2%
Manganese	Asp	Oδ2	4.3	0.4	31.5%
Manganese	Glu	Oε1	2.1	0.1	100.0%
Manganese	Glu	Oε2	3.4	0.5	71.1%
Manganese	Glu	Oε2	4.3	0.4	28.9%
Manganese	His	Nε2	2.19	0.06	97.1%
Manganese	His	Nε2	2.40	0.06	2.9%
Iron	Asp	Oδ1	2.1	0.2	100.0%
Iron	Asp	Oδ2	2.4	0.5	37.2%
Iron	Asp	Oδ2	3.4	0.5	62.8%
Iron	Cys	Sγ	2.32	0.06	97.3%

Iron	Cys	Sγ	2.53	0.06	2.7%
Iron	Glu	Oε1	1.99	0.08	100.0%
Iron	Glu	Oε2	3.5	0.3	100.0%
Iron	His	Nε2	2.0	0.1	64.7%
Iron	His	Nε2	2.2	0.1	35.3%
Iron	Met	Sδ	2.32	0.8	100.0%
Iron	Tyr	OH	1.99	0.07	95.9%
Iron	Tyr	OH	2.23	0.07	4.1%
Nickel	Asp	Oδ1	2.04	0.06	5.5%
Nickel	Asp	Oδ1	2.20	0.06	94.5%
Nickel	Asp	Oδ2	4.4	0.2	100.0%
Nickel	His	Nδ1	1.9	0.1	9.1%
Nickel	His	Nδ1	2.0	0.1	8.8%
Nickel	His	Nδ1	2.2	0.1	82.1%
Nickel	His	Nε2	2.1	0.1	25.4%
Nickel	His	Nε2	2.3	0.1	74.6%
Copper	Cys	Sγ	2.2	0.1	97.0%
Copper	Cys	Sγ	2.4	0.1	2.9%
Copper	His	Nδ1	2.0	0.1	100.0%
Copper	His	Nε2	2.03	0.07	95.7%
Copper	His	Nε2	2.25	0.07	4.3%
Copper	Met	Sδ	2.5	0.2	78.0%
Copper	Met	Sδ	2.9	0.2	22.0%
Zinc(II)	Asp	Oδ1	2.0	0.1	100.0%
Zinc(II)	Asp	Oδ2	2.9	0.3	79.5%
Zinc(II)	Asp	Oδ2	4.2	0.4	20.5%
Zinc(II)	Cys	Sγ	2.32	0.04	100.0%

Zinc(II)	Glu	Oε1	2.0	0.1	100.0%
Zinc(II)	Glu	Oε2	3.0	0.3	100.0%
Zinc(II)	His	Nδ1	2.05	0.06	97.6%
Zinc(II)	His	Nδ1	2.27	0.06	2.4%
Zinc(II)	His	Nε2	2.03	0.07	100.0%

Table S3 For each metal-donor atom (DA) pair, the center of the peak(s) with a density higher than 0.2 was computed. In addition, the integral of each peak is also reported to give information on the contribution of each peak to the distribution. These values were computed considering only dinuclear sites in the highest resolution range, i.e. < 1.5 Å.

**Dinuclear sites, resolution range < 1.5 Å**					
**Metal**	**Residue**	**DA**	**Peak (Å)**	**Std (Å)**	**Peak contribution**
Sodium(I)	Asp	Oδ1	2.5	0.2	100.0%
Sodium(I)	Asp	Oδ2	3.7	0.5	41.0%
Sodium(I)	Asp	Oδ2	4.7	0.4	59.0%
Potassium(I)	Asp	O	2.6	0.1	47.30%
Potassium(I)	Asp	O	2.8	0.1	52.7%
Magnesium(II)	Asp	Oδ1	2.1	0.1	95.6%
Magnesium(II)	Asp	Oδ1	2.5	0.1	4.4%
Magnesium(II)	Asp	Oδ2	3.4	0.3	74.7%
Magnesium(II)	Asp	Oδ2	4.2	0.3	25.3%
Magnesium(II)	Glu	Oε1	2.1	0.2	100.0%
Magnesium(II)	Glu	Oε2	3.5	0.4	100.0%
Calcium(II)	Asn	Oδ1	2.34	0.05	77.9%
Calcium(II)	Asn	Oδ1	2.43	0.05	22.2%
Calcium(II)	Asp	Oδ1	2.36	0.08	100.0%
Calcium(II)	Asp	Oδ2	2.6	0.4	26.5%
Calcium(II)	Asp	Oδ2	3.8	0.4	38.5%
Calcium(II)	Asp	Oδ2	4.3	0.3	35.0%
Calcium(II)	Glu	O	2.34	0.04	100.0%
Calcium(II)	Glu	Oε1	2.41	0.09	100.0%
Calcium(II)	Glu	Oε2	2.6	0.2	57.7%
Calcium(II)	Glu	Oε2	4.4	0.3	42.3%

Calcium(II)	Gly	O	2.30	0.07	42.9%
Calcium(II)	Gly	O	2.42	0.07	57.1%
Manganese	Asp	Oδ1	2.2	0.1	100.0%
Manganese	Asp	Oδ2	2.5	0.4	13.3%
Manganese	Asp	Oδ2	3.5	0.5	58.5%
Manganese	Asp	Oδ2	4.3	0.4	28.2%
Manganese	Glu	Oε1	1.9	0.1	8.6%
Manganese	Glu	Oε1	2.1	0.1	91.4%
Manganese	Glu	Oε2	3.4	0.2	100.0%
Manganese	His	Nδ1	2.23	0.06	50.0%
Manganese	His	Nδ1	2.26	0.06	50.0%
Manganese	His	Nε2	2.2	0.1	100.0%
Iron	Asp	Oδ1	2.11	0.08	79.9%
Iron	Asp	Oδ1	2.29	0.08	20.1%
Iron	Asp	Oδ2	3.4	0.3	100.0%
Iron	Cys	Sγ	2.30	0.05	100.0%
Iron	Glu	Oε1	2.1	0.1	93.5%
Iron	Glu	Oε1	2.5	0.1	6.5%
Iron	Glu	Oε2	2.3	0.5	25.2%
Iron	Glu	Oε2	3.4	0.3	74.8%
Iron	His	Nδ1	2.1	0.1	62.2%
Iron	His	Nδ1	2.24	0.07	37.9%
Iron	His	Nε2	2.18	0.07	100.0%
Copper	His	Nε2	2.1	0.1	93.5%
Copper	His	Nε2	2.4	0.1	6.5%
Zinc(II)	Asp	Oδ1	2.0	0.1	94.7%

Zinc(II)	Asp	Oδ1	2.6	0.1	5.33%
Zinc(II)	Asp	Oδ2	3.4	0.3	100.0%
Zinc(II)	Cys	Sγ	2.30	0.05	100.0%
Zinc(II)	Glu	Oε1	2.0	0.2	100.0%
Zinc(II)	Glu	Oε2	2.6	0.4	100.0%
Zinc(II)	His	Nδ1	2.1	0.1	96.4%
Zinc(II)	His	Nδ1	2.4	0.1	3.7%
Zinc(II)	His	Nε2	2.05	0.07	100.0%

Table S4 For each metal-donor atom (DA) pair, the center of the peak(s) with a density higher than 0.2 was computed. In addition, the integral of each peak is also reported to give information on the contribution of each peak to the distribution. These values were computed considering only trinuclear sites in the highest resolution range, i.e. < 1.5 Å.

**Trinuclear sites, resolution range < 1.5 Å**					
**Metal**	**Residue**	**DA**	**Peak (Å)**	**Std (Å)**	**Peak contribution**
Magnesium(II)	Asp	Oδ1	2.1	0.1	100.0%
Magnesium(II)	Asp	Oδ2	3.5	0.4	57.9%
Magnesium(II)	Asp	Oδ2	4.1	0.4	42.1%
Magnesium(II)	Glu	Oε1	2.1	0.3	77.9%
Magnesium(II)	Glu	Oε1	2.7	0.3	22.1%
Magnesium(II)	Glu	Oε2	3.5	0.3	100.0%
Calcium(II)	Asn	Oδ1	2.39	0.06	100.0%
Calcium(II)	Asp	Oδ1	2.35	0.09	68.3%
Calcium(II)	Asp	Oδ1	2.5	0.1	31.7%
Calcium(II)	Asp	Oδ2	2.7	0.3	39.6%
Calcium(II)	Asp	Oδ2	4.3	0.3	60.4%
Calcium(II)	Glu	Oε1	2.4	0.1	100.0%
Calcium(II)	Glu	Oε2	2.6	0.3	32.1%
Calcium(II)	Glu	Oε2	4.0	0.4	68.0%
Iron	Cys	Sγ	2.31	0.02	100.0%
Copper	His	Nε2	1.96	0.07	46.7%
Copper	His	Nε2	2.03	0.07	53.3%
Zinc(II)	Asp	Oδ1	2.0	0.2	100.0%
Zinc(II)	Asp	Oδ2	3.2	0.4	100.0%
Zinc(II)	Cys	Sγ	2.36	0.03	100.0%
Zinc(II)	His	Nε2	2.06	0.06	96.6%
Zinc(II)	His	Nε2	2.28	0.06	3.4%

Table S5 For each DA-transition metal pair, the center of the peak(s) with a density higher than 0.2 was computed. In addition, the integral of each peak is also reported to give information on the contribution of each peak to the distribution. These values were computed considering only mononuclear sites in the 1.5-2 Å resolution range.

**Mononuclear sites, resolution range 1.5-2 Å**					
**Metal**	**Residue**	**DA**	**Peak (Å)**	**Std (Å)**	**Peak contribution**
Sodium(I)	Ala	O	2.3	0.2	100.0%
Sodium(I)	Arg	O	2.4	0.2	100.0%
Sodium(I)	Asn	O	2.3	0.2	86.1%
Sodium(I)	Asn	O	2.7	0.2	13.9%
Sodium(I)	Asn	Oδ1	2.4	0.2	100.0%
Sodium(I)	Asp	O	2.4	0.2	77.3%
Sodium(I)	Asp	O	2.8	0.2	22.7%
Sodium(I)	Asp	Oδ1	2.4	0.2	77.5%
Sodium(I)	Asp	Oδ1	2.7	0.2	22.5%
Sodium(I)	Asp	Oδ2	3.7	0.5	43.5%
Sodium(I)	Asp	Oδ2	4.4	0.4	56.6%
Sodium(I)	Cys	O	2.4	0.2	100.0%
Sodium(I)	Gln	O	2.4	0.2	81.9%
Sodium(I)	Gln	O	2.7	0.2	18.1%
Sodium(I)	Gln	Oε1	2.	0.2	100.0%
Sodium(I)	Glu	O	2.4	0.2	66.8%
Sodium(I)	Glu	O	2.8	0.2	33.3%
Sodium(I)	Glu	Oε1	2.4	0.3	56.5%
Sodium(I)	Glu	Oε1	2.7	0.2	43.5%
Sodium(I)	Glu	Oε2	3.8	0.5	50.7%
Sodium(I)	Glu	Oε2	4.5	0.4	49.3%

Sodium(I)	Gly	O	2.4	0.2	88.5%
Sodium(I)	Gly	O	2.9	0.2	11.5%
Sodium(I)	Ile	O	2.4	0.2	100.0%
Sodium(I)	Leu	O	2.4	0.2	100.0%
Sodium(I)	Lys	O	2.4	0.1	100.0%
Sodium(I)	Phe	O	2.4	0.2	90.6%
Sodium(I)	Phe	O	2.8	0.2	9.4%
Sodium(I)	Pro	O	2.4	0.2	100.0%
Sodium(I)	Ser	O	2.4	0.2	85.8%
Sodium(I)	Ser	O	2.7	0.2	14.2%
Sodium(I)	Ser	Oγ	2.5	0.2	69.1%
Sodium(I)	Ser	Oγ	2.8	0.2	30.9%
Sodium(I)	Thr	O	2.4	0.2	74.2%
Sodium(I)	Thr	O	2.7	0.2	25.8%
Sodium(I)	Thr	Oγ1	2.4	0.2	100.0%
Sodium(I)	Tyr	O	2.4	0.2	86.9%
Sodium(I)	Tyr	O	2.9	0.2	13.1%
Sodium(I)	Val	O	2.3	0.2	100.0%
Potassium(I)	Ala	O	2.7	0.2	59.0%
Potassium(I)	Ala	O	2.9	0.2	41.0%
Potassium(I)	Asn	Oδ1	2.7	0.2	100.0%
Potassium(I)	Asp	O	2.4	0.2	13.0%
Potassium(I)	Asp	O	2.9	0.2	87.1%
Potassium(I)	Asp	Oδ1	2.8	0.2	100.0%
Potassium(I)	Asp	Oδ2	4.2	0.4	100.0%
Potassium(I)	Glu	Oε1	2.7	0.2	100.0%
Potassium(I)	Glu	Oε2	3.7	0.6	32.5%

Potassium(I)	Glu	Oε2	4.7	0.4	67.5%
Potassium(I)	Gly	O	2.7	0.1	100.0%
Potassium(I)	Leu	O	2.7	0.1	100.0%
Potassium(I)	Ser	O	2.4	0.2	12.2%
Potassium(I)	Ser	O	2.8	0.2	87.8%
Potassium(I)	Ser	Oγ	2.7	0.2	47.4%
Potassium(I)	Ser	Oγ	2.9	0.2	52.6%
Potassium(I)	Thr	O	2.8	0.1	100.0%
Potassium(I)	Val	O	2.6	0.1	100.0%
Magnesium(II)	Ala	O	2.3	0.2	100.0%
Magnesium(II)	Arg	O	2.5	0.2	100.0%
Magnesium(II)	Asn	O	2.3	0.2	66.0%
Magnesium(II)	Asn	O	2.7	0.2	34.0%
Magnesium(II)	Asn	Oδ1	2.1	0.2	100.0%
Magnesium(II)	Asp	O	2.2	0.2	88.3%
Magnesium(II)	Asp	O	2.7	0.2	11.7%
Magnesium(II)	Asp	Oδ1	2.1	0.2	100.0%
Magnesium(II)	Asp	Oδ2	3.4	0.4	71.2%
Magnesium(II)	Asp	Oδ2	4.2	0.4	28.8%
Magnesium(II)	Gln	Oε1	2.2	0.2	89.6%
Magnesium(II)	Gln	Oε1	2.9	0.2	10.4%
Magnesium(II)	Glu	Oε1	2.1	0.2	100.0%
Magnesium(II)	Glu	Oε2	3.4	0.4	60.8%
Magnesium(II)	Glu	Oε2	3.9	0.4	39.2%
Magnesium(II)	Gly	O	2.1	0.2	100.0%
Magnesium(II)	His	Nδ1	2.2	0.2	100.0%
Magnesium(II)	His	Nε2	2.2	0.2	100.0%

Magnesium(II)	Ile	O	2.3	0.2	100.0%
Magnesium(II)	Leu	O	2.3	0.3	52.7%
Magnesium(II)	Leu	O	2.5	0.3	47.3%
Magnesium(II)	Ser	O	2.2	0.3	100.0%
Magnesium(II)	Ser	Oγ	2.1	0.2	94.0%
Magnesium(II)	Ser	Oγ	2.7	0.2	6.0%
Magnesium(II)	Thr	O	2.2	0.2	100.0%
Magnesium(II)	Thr	Oγ1	2.1	0.2	95.0%
Magnesium(II)	Thr	Oγ1	2.5	0.2	5.0%
Magnesium(II)	Val	O	2.3	0.2	85.9%
Magnesium(II)	Val	O	2.7	0.2	14.1%
Calcium(II)	Ala	O	2.3	0.2	88.9%
Calcium(II)	Ala	O	2.9	0.2	11.1%
Calcium(II)	Arg	O	2.4	0.2	83.3%
Calcium(II)	Arg	O	2.9	0.2	16.7%
Calcium(II)	Asn	O	2.3	0.1	100.0%
Calcium(II)	Asn	Oδ1	2.4	0.1	100.0%
Calcium(II)	Asp	O	2.4	0.1	100.0%
Calcium(II)	Asp	Oδ1	2.4	0.2	92.6%
Calcium(II)	Asp	Oδ1	2.8	0.2	7.4%
Calcium(II)	Asp	Oδ2	2.6	0.4	23.1%
Calcium(II)	Asp	Oδ2	3.8	0.4	42.6%
Calcium(II)	Asp	Oδ2	4.3	0.3	34.3%
Calcium(II)	Gln	O	2.3	0.1	100.0%
Calcium(II)	Gln	Oε1	2.4	0.1	100.0%
Calcium(II)	Glu	O	2.3	0.2	100.0%
Calcium(II)	Glu	Oε1	2.4	0.2	93.8%

Calcium(II)	Glu	Oε1	2.8	0.2	6.2%
Calcium(II)	Glu	Oε2	2.6	0.3	42.6%
Calcium(II)	Glu	Oε2	3.8	0.5	26.2%
Calcium(II)	Glu	Oε2	4.5	0.4	31.3%
Calcium(II)	Gly	O	2.3	0.2	100.0%
Calcium(II)	His	O	2.4	0.1	100.0%
Calcium(II)	Ile	O	2.3	0.1	100.0%
Calcium(II)	Leu	O	2.3	0.1	100.0%
Calcium(II)	Lys	O	2.3	0.1	90.9%
Calcium(II)	Lys	O	2.7	0.1	9.1%
Calcium(II)	Phe	O	2.3	0.1	95.8%
Calcium(II)	Phe	O	2.7	0.1	4.2%
Calcium(II)	Pro	O	2.4	0.2	100.0%
Calcium(II)	Ser	O	2.4	0.1	100.0%
Calcium(II)	Ser	Oγ	2.5	0.2	91.4%
Calcium(II)	Ser	Oγ	2.9	0.2	8.6%
Calcium(II)	Thr	O	2.4	0.2	87.3%
Calcium(II)	Thr	O	2.8	0.2	12.7%
Calcium(II)	Thr	Oγ1	2.5	0.1	100.0%
Calcium(II)	Tyr	O	2.3	0.1	100.0%
Calcium(II)	Val	O	2.3	0.1	100.0%
Manganese	Asp	Oδ1	2.2	0.2	100.0%
Manganese	Asp	Oδ2	2.5	0.4	11.1%
Manganese	Asp	Oδ2	3.5	0.4	59.3%
Manganese	Asp	Oδ2	4.2	0.4	29.6%
Manganese	Glu	Oε1	2.1	0.2	100.0%
Manganese	Glu	Oε2	2.4	0.4	13.3%

Manganese	Glu	Oε2	3.5	0.4	69.1%
Manganese	Glu	Oε2	4.2	0.4	17.6%
Manganese	His	Nε2	2.2	0.1	100.0%
Iron	Asp	Oδ1	2.0	0.2	44.2%
Iron	Asp	Oδ1	2.1	0.2	55.8%
Iron	Asp	Oδ2	2.6	0.4	27.9%
Iron	Asp	Oδ2	3.5	0.5	52.0%
Iron	Asp	Oδ2	4.1	0.3	20.1%
Iron	Cys	Sγ	2.3	0.1	100.0%
Iron	Glu	Oε1	2.1	0.2	100.0%
Iron	Glu	Oε2	2.4	0.5	25.7%
Iron	Glu	Oε2	3.4	0.4	74.3%
Iron	His	Nε2	2.1	0.1	50.0%
Iron	His	Nε2	2.1	0.1	50.0%
Iron	Met	Sδ	2.3	0.1	91.1%
Iron	Met	Sδ	2.6	0.1	8.9%
Iron	Tyr	OH	2.0	0.2	100.0%
Nickel	Asp	Oδ1	2.1	0.2	100.0%
Nickel	Asp	Oδ2	3.3	0.4	39.5%
Nickel	Asp	Oδ2	4.4	0.3	60.5%
Nickel	His	Nδ1	2.3	0.2	100.0%
Nickel	His	Nε2	2.1	0.1	52.8%
Nickel	His	Nε2	2.2	0.1	47.2%
Copper	Cys	Sγ	2.19	0.07	100.0%
Copper	His	Nδ1	2.1	0.1	100.0%
Copper	His	Nε2	2.04	0.09	100.0%
Copper	Met	Sδ	2.5	0.2	100.0%

Zinc(II)	Asp	Oδ1	2.0	0.2	100.0%
Zinc(II)	Asp	Oδ2	2.9	0.3	100.0%
Zinc(II)	Cys	Sγ	2.3	0.1	100.0%
Zinc(II)	Glu	Oε1	2.0	0.2	100.0%
Zinc(II)	Glu	Oε2	2.9	0.4	75.4%
Zinc(II)	Glu	Oε2	4.2	0.4	24.6%
Zinc(II)	His	Nδ1	2.1	0.1	100.0%
Zinc(II)	His	Nε2	2.1	0.1	100.0%

Table S6 For each metal-donor atom (DA) pair, the center of the peak(s) with a density higher than 0.2 was computed. In addition, the integral of each peak is also reported to give information on the contribution of each peak to the distribution. These values were computed considering only mononuclear sites in the 2-2.5 Å resolution range.

**Mononuclear sites, resolution range 2-2.5 Å**					
**Metal**	**Residue**	**DA**	**Peak (Å)**	**Std (Å)**	**Peak contribution**
Sodium(I)	Ala	O	2.4	0.2	100.0%
Sodium(I)	Arg	O	2.3	0.2	29.9%
Sodium(I)	Arg	O	2.5	0.2	33.6%
Sodium(I)	Arg	O	2.7	0.2	36.5%
Sodium(I)	Asn	O	2.4	0.2	100.0%
Sodium(I)	Asn	Oδ1	2.4	0.2	76.0%
Sodium(I)	Asn	Oδ1	2.7	0.2	24.0%
Sodium(I)	Asp	O	2.4	0.2	76.1%
Sodium(I)	Asp	O	2.8	0.2	23.9%
Sodium(I)	Asp	Oδ1	2.4	0.2	100.0%
Sodium(I)	Asp	Oδ2	3.8	0.5	43.5%
Sodium(I)	Asp	Oδ2	4.3	0.4	56.5%
Sodium(I)	Cys	O	2.3	0.2	100.0%
Sodium(I)	Gln	O	2.4	0.2	100.0%
Sodium(I)	Gln	Oε1	2.4	0.2	82.3%
Sodium(I)	Gln	Oε1	2.9	0.2	17.7%
Sodium(I)	Glu	O	2.4	0.2	100.0%
Sodium(I)	Glu	Oε1	2.4	0.2	65.5%
Sodium(I)	Glu	Oε1	2.8	0.2	34.6%
Sodium(I)	Glu	Oε2	3.5	0.5	62.6%
Sodium(I)	Glu	Oε2	4.5	0.5	37.4%

Sodium(I)	Gly	O	2.4	0.2	100.0%
Sodium(I)	Ile	O	2.4	0.2	100.0%
Sodium(I)	Leu	O	2.4	0.2	100.0%
Sodium(I)	Lys	O	2.4	0.2	100.0%
Sodium(I)	Phe	O	2.4	0.2	100.0%
Sodium(I)	Pro	O	2.7	0.2	100.0%
Sodium(I)	Ser	O	2.4	0.2	100.0%
Sodium(I)	Ser	Oγ	2.3	0.2	51.8%
Sodium(I)	Ser	Oγ	2.6	0.2	48.2%
Sodium(I)	Thr	O	2.6	0.2	66.6%
Sodium(I)	Thr	O	2.7	0.2	33.4%
Sodium(I)	Thr	Oγ1	2.3	0.2	41.7%
Sodium(I)	Thr	Oγ1	2.6	0.2	31.2%
Sodium(I)	Thr	Oγ1	2.8	0.2	27.1%
Sodium(I)	Tyr	O	2.4	0.2	100.0%
Sodium(I)	Val	O	2.4	0.2	100.0%
Potassium(I)	Ala	O	2.7	0.1	52.2%
Potassium(I)	Ala	O	2.9	0.1	47.8%
Potassium(I)	Asn	Oδ1	2.8	0.1	55.7%
Potassium(I)	Asn	Oδ1	2.9	0.14	44.3%
Potassium(I)	Asp	O	2.8	0.15	100.0%
Potassium(I)	Asp	Oδ1	2.8	0.18	100.0%
Potassium(I)	Asp	Oδ2	4.6	0.37	100.0%
Potassium(I)	Glu	Oε1	2.7	0.19	100.0%
Potassium(I)	Glu	Oε2	3.6	0.56	33.2%
Potassium(I)	Glu	Oε2	4.6	0.38	66.8%
Potassium(I)	Gly	O	2.8	0.13	100.0%

Potassium(I)	Leu	O	2.4	0.15	10.4%
Potassium(I)	Leu	O	2.8	0.15	89.6%
Potassium(I)	Ser	O	2.8	0.15	100.0%
Potassium(I)	Ser	Oγ	2.7	0.13	52.0%
Potassium(I)	Ser	Oγ	2.9	0.13	48.0%
Potassium(I)	Thr	O	2.7	0.14	37.6%
Potassium(I)	Thr	O	2.8	0.14	62.4%
Potassium(I)	Val	O	2.7	0.14	100.0%
Magnesium(II)	Ala	O	2.3	0.25	100.0%
Magnesium(II)	Arg	O	2.3	0.25	100.0%
Magnesium(II)	Asn	O	2.3	0.24	70.5%
Magnesium(II)	Asn	O	2.7	0.24	29.5%
Magnesium(II)	Asn	Oδ1	2.2	0.23	100.0%
Magnesium(II)	Asp	O	2.2	0.22	85.7%
Magnesium(II)	Asp	O	2.7	0.22	14.3%
Magnesium(II)	Asp	Oδ1	2.1	0.25	100.0%
Magnesium(II)	Asp	Oδ2	3.5	0.43	58.9%
Magnesium(II)	Asp	Oδ2	4.2	0.4	41.1%
Magnesium(II)	Gln	Oε1	2.2	0.27	80.4%
Magnesium(II)	Gln	Oε1	2.9	0.27	19.6%
Magnesium(II)	Glu	Oε1	2.1	0.26	88.9%
Magnesium(II)	Glu	Oε1	2.8	0.26	11.1%
Magnesium(II)	Glu	Oε2	3.6	0.41	100.0%
Magnesium(II)	Gly	O	2.2	0.3	66.9%
Magnesium(II)	Gly	O	2.6	0.29	33.1%
Magnesium(II)	His	Nδ1	2.2	0.22	85.1%
Magnesium(II)	His	Nδ1	2.8	0.22	14.9%

Magnesium(II)	His	Nε2	2.2	0.18	100.0%
Magnesium(II)	Ile	O	2.2	0.27	51.1%
Magnesium(II)	Ile	O	2.5	0.27	48.9%
Magnesium(II)	Leu	O	2.2	0.26	53.5%
Magnesium(II)	Leu	O	2.5	0.26	46.5%
Magnesium(II)	Ser	O	2.3	0.21	100.0%
Magnesium(II)	Ser	Oγ	2.2	0.27	80.2%
Magnesium(II)	Ser	Oγ	2.8	0.26	19.8%
Magnesium(II)	Thr	O	2.3	0.25	100.0%
Magnesium(II)	Thr	Oγ1	2.1	0.25	100.0%
Magnesium(II)	Val	O	2.3	0.22	100.0%
Calcium(II)	Ala	O	2.3	0.18	100.0%
Calcium(II)	Arg	O	2.3	0.24	70.9%
Calcium(II)	Arg	O	2.8	0.24	29.1%
Calcium(II)	Asn	O	2.4	0.18	80.4%
Calcium(II)	Asn	O	2.7	0.18	19.6%
Calcium(II)	Asn	Oδ1	2.4	0.17	100.0%
Calcium(II)	Asp	O	2.4	0.17	100.0%
Calcium(II)	Asp	Oδ1	2.4	0.17	100.0%
Calcium(II)	Asp	Oδ2	2.6	0.4	26.6%
Calcium(II)	Asp	Oδ2	3.8	0.47	35.4%
Calcium(II)	Asp	Oδ2	4.3	0.37	38.0%
Calcium(II)	Gln	O	2.3	0.19	89.6%
Calcium(II)	Gln	O	2.8	0.19	10.4%
Calcium(II)	Gln	Oε1	2.4	0.18	100.0%
Calcium(II)	Glu	O	2.3	0.17	90.0%
Calcium(II)	Glu	O	2.8	0.17	10.0%

Calcium(II)	Glu	Oε1	2.4	0.18	100.0%
Calcium(II)	Glu	Oε2	2.6	0.3	64.2%
Calcium(II)	Glu	Oε2	4.4	0.4	35.9%
Calcium(II)	Gly	O	2.4	0.18	92.6%
Calcium(II)	Gly	O	2.9	0.17	7.4%
Calcium(II)	His	O	2.4	0.13	100.0%
Calcium(II)	Ile	O	2.3	0.17	100.0%
Calcium(II)	Leu	O	2.4	0.18	100.0%
Calcium(II)	Lys	O	2.3	0.16	100.0%
Calcium(II)	Phe	O	2.4	0.18	92.1%
Calcium(II)	Phe	O	2.9	0.18	7.9%
Calcium(II)	Pro	O	2.4	0.16	100.0%
Calcium(II)	Ser	O	2.4	0.17	93.7%
Calcium(II)	Ser	O	2.9	0.17	6.3%
Calcium(II)	Ser	Oγ	2.5	0.19	100.0%
Calcium(II)	Thr	O	2.4	0.17	100.0%
Calcium(II)	Thr	Oγ1	2.5	0.17	100.0%
Calcium(II)	Tyr	O	2.3	0.16	87.7%
Calcium(II)	Tyr	O	2.7	0.16	12.3%
Calcium(II)	Val	O	2.3	0.19	100.0%
Manganese	Asp	Oδ1	2.2	0.19	100.0%
Manganese	Asp	Oδ2	3.5	0.48	61.0%
Manganese	Asp	Oδ2	4.3	0.4	39.0%
Manganese	Glu	Oε1	2.2	0.2	100.0%
Manganese	Glu	Oε2	3.5	0.42	79.9%
Manganese	Glu	Oε2	4.4	0.35	20.1%
Manganese	His	Nε2	2.2	0.16	100.0%

Iron	Asp	Oδ1	2.1	0.25	100.0%
Iron	Asp	Oδ2	2.7	0.41	27.0%
Iron	Asp	Oδ2	3.4	0.55	44.8%
Iron	Asp	Oδ2	4.2	0.39	28.2%
Iron	Cys	Sγ	2.3	0.13	100.0%
Iron	Glu	Oε1	2.1	0.27	79.2%
Iron	Glu	Oε1	2.6	0.3	20.8%
Iron	Glu	Oε2	2.4	0.4	26.8%
Iron	Glu	Oε2	3.4	0.4	73.2%
Iron	His	Nε2	2.1	0.2	100.0%
Iron	Met	Sδ	2.3	0.1	100.0%
Iron	Tyr	OH	1.9	0.2	100.0%
Nickel	Asp	Oδ1	2.2	0.2	100.0%
Nickel	Asp	Oδ2	3.3	0.4	84.3%
Nickel	Asp	Oδ2	4.3	0.4	15.7%
Nickel	His	Nδ1	2.1	0.3	100.0%
Nickel	His	Nε2	2.2	0.2	100.0%
Copper	Cys	Sγ	2.2	0.1	100.0%
Copper	His	Nδ1	2.0	0.2	100.0%
Copper	His	Nε2	2.1	0.1	93.7%
Copper	His	Nε2	2.5	0.1	6.3%
Copper	Met	Sδ	2.5	0.2	63.8%
Copper	Met	Sδ	2.9	0.2	36.2%
Zinc(II)	Asp	Oδ1	2.0	0.2	100.0%
Zinc(II)	Asp	Oδ2	3.1	0.4	74.1%
Zinc(II)	Asp	Oδ2	4.1	0.4	25.9%
Zinc(II)	Cys	Sγ	2.3	0.1	100.0%

Zinc(II)	Glu	Oε1	2.0	0.2	100.0%
Zinc(II)	Glu	Oε2	2.9	0.4	73.5%
Zinc(II)	Glu	Oε2	4.1	0.5	26.6%
Zinc(II)	His	Nδ1	2.1	0.2	100.0%
Zinc(II)	His	Nε2	2.1	0.2	100.0%

Table S7 For each metal-donor atom (DA) pair, the center of the peak(s) with a density higher than 0.2 was computed. In addition, the integral of each peak is also reported to give information on the contribution of each peak to the distribution. These values were computed considering only mononuclear sites in the 2.5-3 Å resolution range.

Mononuclear sites, resolution range 2.5-3 Å					
**Metal**	**Residue**	**DA**	**Peak (Å)**	**Std (Å)**	**Peak contribution**
Sodium(I)	Ala	O	2.4	0.2	100.0%
Sodium(I)	Arg	O	2.8	0.2	100.0%
Sodium(I)	Asn	O	2.5	0.3	39.5%
Sodium(I)	Asn	O	2.8	0.3	60.5%
Sodium(I)	Asn	Oδ1	2.4	0.2	68.4%
Sodium(I)	Asn	Oδ1	2.8	0.2	31.6%
Sodium(I)	Asp	O	2.6	0.2	100.0%
Sodium(I)	Asp	Oδ1	2.4	0.2	100.0%
Sodium(I)	Asp	Oδ2	3.7	0.5	49.7%
Sodium(I)	Asp	Oδ2	4.4	0.4	50.3%
Sodium(I)	Cys	O	2.4	0.2	63.6%
Sodium(I)	Cys	O	2.7	0.2	36.4%
Sodium(I)	Gln	O	2.4	0.2	52.2%
Sodium(I)	Gln	O	2.8	0.2	47.8%
Sodium(I)	Gln	Oε1	2.5	0.2	100.0%
Sodium(I)	Glu	O	2.6	0.2	100.0%
Sodium(I)	Glu	Oε1	2.4	0.2	59.2%
Sodium(I)	Glu	Oε1	2.7	0.2	40.8%
Sodium(I)	Glu	Oε2	3.5	0.5	100.0%
Sodium(I)	Gly	O	2.3	0.2	44.1%
Sodium(I)	Gly	O	2.6	0.2	55.9%
Sodium(I)	Ile	O	2.5	0.2	100.0%
Sodium(I)	Leu	O	2.4	0.2	51.7%

Sodium(I)	Leu	O	2.7	0.2	48.3%
Sodium(I)	Lys	O	2.7	0.2	100.0%
Sodium(I)	Phe	O	2.4	0.2	76.7%
Sodium(I)	Phe	O	2.9	0.2	23.4%
Sodium(I)	Pro	O	2.7	0.2	100.0%
Sodium(I)	Ser	O	2.4	0.2	69.6%
Sodium(I)	Ser	O	2.8	0.2	30.5%
Sodium(I)	Ser	Oγ	2.4	0.2	50.9%
Sodium(I)	Ser	Oγ	2.8	0.22	49.1%
Sodium(I)	Thr	O	2.4	0.26	71.6%
Sodium(I)	Thr	O	2.9	0.26	28.4%
Sodium(I)	Thr	Oγ1	2.5	0.23	51.9%
Sodium(I)	Thr	Oγ1	2.8	0.23	48.1%
Sodium(I)	Tyr	O	2.6	0.17	100.0%
Sodium(I)	Val	O	2.4	0.23	100.0%
Potassium(I)	Ala	O	2.9	0.14	100.0%
Potassium(I)	Asn	Oδ1	2.8	0.16	100.0%
Potassium(I)	Asp	O	2.9	0.16	100.0%
Potassium(I)	Asp	Oδ1	2.6	0.17	58.3%
Potassium(I)	Asp	Oδ1	2.8	0.17	41.7%
Potassium(I)	Asp	Oδ2	2.7	0.35	28.4%
Potassium(I)	Asp	Oδ2	4.4	0.33	71.6%
Potassium(I)	Glu	Oε1	2.7	0.14	100.0%
Potassium(I)	Glu	Oε2	3.2	0.43	20.1%
Potassium(I)	Glu	Oε2	4.8	0.3	79.9%
Potassium(I)	Gly	O	2.8	0.12	100.0%
Potassium(I)	Leu	O	2.9	0.2	100.0%
Potassium(I)	Ser	O	2.7	0.15	100.0%
Potassium(I)	Ser	Oγ	2.9	0.12	100.0%

Potassium(I)	Thr	O	2.8	0.15	100.0%
Potassium(I)	Val	O	2.7	0.14	100.0%
Magnesium(II)	Ala	O	2.4	0.24	69.4%
Magnesium(II)	Ala	O	2.8	0.23	30.7%
Magnesium(II)	Arg	O	2.7	0.21	100.0%
Magnesium(II)	Asn	O	2.2	0.27	100.0%
Magnesium(II)	Asn	Oδ1	2.2	0.27	100.0%
Magnesium(II)	Asp	O	2.2	0.24	82.6%
Magnesium(II)	Asp	O	2.8	0.24	17.5%
Magnesium(II)	Asp	Oδ1	2.1	0.29	49.9%
Magnesium(II)	Asp	Oδ1	2.3	0.3	50.2%
Magnesium(II)	Asp	Oδ2	3.5	0.5	53.2%
Magnesium(II)	Asp	Oδ2	4.1	0.5	46.8%
Magnesium(II)	Gln	Oε1	2.3	0.3	63.3%
Magnesium(II)	Gln	Oε1	2.8	0.3	36.7%
Magnesium(II)	Glu	Oε1	2.1	0.3	71.1%
Magnesium(II)	Glu	Oε1	2.7	0.3	28.9%
Magnesium(II)	Glu	Oε2	3.8	0.5	100.0%
Magnesium(II)	Gly	O	2.3	0.3	48.8%
Magnesium(II)	Gly	O	2.6	0.3	51.2%
Magnesium(II)	His	Nδ1	2.2	0.3	27.7%
Magnesium(II)	His	Nδ1	2.4	0.3	52.7%
Magnesium(II)	His	Nδ1	2.8	0.3	19.7%
Magnesium(II)	His	Nε2	2.2	0.3	70.2%
Magnesium(II)	His	Nε2	2.8	0.3	29.8%
Magnesium(II)	Ile	O	2.2	0.3	51.5%
Magnesium(II)	Ile	O	2.6	0.3	48.5%
Magnesium(II)	Leu	O	2.2	0.3	37.1%
Magnesium(II)	Leu	O	2.9	0.3	62.9%

Magnesium(II)	Ser	O	2.6	0.2	100.0%
Magnesium(II)	Ser	Oγ	2.1	0.3	100.0%
Magnesium(II)	Thr	O	2.7	0.3	100.0%
Magnesium(II)	Thr	Oγ1	2.2	0.3	58.1%
Magnesium(II)	Thr	Oγ1	2.6	0.3	41.9%
Magnesium(II)	Val	O	2.3	0.3	46.2%
Magnesium(II)	Val	O	2.6	0.3	53.8%
Calcium(II)	Ala	O	2.4	0.2	100.0%
Calcium(II)	Arg	O	2.3	0.2	100.0%
Calcium(II)	Asn	O	2.4	0.2	100.0%
Calcium(II)	Asn	Oδ1	2.4	0.2	100.0%
Calcium(II)	Asp	O	2.4	0.2	100.0%
Calcium(II)	Asp	Oδ1	2.4	0.2	100.0%
Calcium(II)	Asp	Oδ2	2.7	0.4	33.7%
Calcium(II)	Asp	Oδ2	4.3	0.4	66.3%
Calcium(II)	Gln	O	2.3	0.2	100.0%
Calcium(II)	Gln	Oε1	2.4	0.3	100.0%
Calcium(II)	Glu	O	2.4	0.2	73.7%
Calcium(II)	Glu	O	2.8	0.2	26.3%
Calcium(II)	Glu	Oε1	2.4	0.2	100.0%
Calcium(II)	Glu	Oε2	2.7	0.4	66.0%
Calcium(II)	Glu	Oε2	4.3	0.5	34.0%
Calcium(II)	Gly	O	2.4	0.2	100.0%
Calcium(II)	His	O	2.4	0.2	100.0%
Calcium(II)	Ile	O	2.4	0.2	100.0%
Calcium(II)	Leu	O	2.4	0.2	100.0%
Calcium(II)	Lys	O	2.3	0.2	100.0%
Calcium(II)	Phe	O	2.4	0.2	100.0%
Calcium(II)	Pro	O	2.4	0.2	100.0%

Calcium(II)	Ser	O	2.4	0.2	100.0%
Calcium(II)	Ser	Oγ	2.4	0.2	68.3%
Calcium(II)	Ser	Oγ	2.8	0.2	31.7%
Calcium(II)	Thr	O	2.4	0.2	100.0%
Calcium(II)	Thr	Oγ1	2.4	0.2	100.0%
Calcium(II)	Tyr	O	2.3	0.2	100.0%
Calcium(II)	Val	O	2.3	0.2	59.6%
Calcium(II)	Val	O	2.5	0.2	40.4%
Manganese	Asp	Oδ1	2.2	0.3	100.0%
Manganese	Asp	Oδ2	3.4	0.5	61.6%
Manganese	Asp	Oδ2	4.2	0.4	38.4%
Manganese	Glu	Oε1	2.2	0.2	100.0%
Manganese	Glu	Oε2	3.2	0.5	63.3%
Manganese	Glu	Oε2	4.0	0.4	36.7%
Manganese	His	Nε2	2.3	0.2	100.0%
Iron	Asp	Oδ1	2.1	0.2	57.5%
Iron	Asp	Oδ1	2.3	0.2	42.5%
Iron	Asp	Oδ2	3.2	0.5	51.4%
Iron	Asp	Oδ2	4.1	0.4	48.6%
Iron	Cys	Sγ	2.3	0.2	100.0%
Iron	Glu	Oε1	2.1	0.2	88.2%
Iron	Glu	Oε1	2.8	0.2	11.8%
Iron	Glu	Oε2	2.4	0.4	36.8%
Iron	Glu	Oε2	3.4	0.4	63.2%
Iron	His	Nε2	2.0	0.2	50.4%
Iron	His	Nε2	2.2	0.2	49.6%
Iron	Met	Sδ	2.3	0.2	84.5%
Iron	Met	Sδ	2.7	0.2	15.5%
Iron	Tyr	OH	2.0	0.2	100.0%

Nickel	Asp	Oδ1	2.1	0.3	49.9%
Nickel	Asp	Oδ1	2.3	0.3	50.1%
Nickel	Asp	Oδ2	3.2	0.4	100.0%
Nickel	His	Nδ1	2.0	0.2	100.0%
Nickel	His	Nε2	2.2	0.2	100.0%
Copper	Cys	Sγ	2.2	0.1	100.0%
Copper	His	Nδ1	2.0	0.2	100.0%
Copper	His	Nε2	2.1	0.2	100.0%
Copper	Met	Sδ	2.4	0.2	100.0%
Zinc(II)	Asp	Oδ1	2.0	0.3	100.0%
Zinc(II)	Asp	Oδ2	3.0	0.4	76.0%
Zinc(II)	Asp	Oδ2	4.0	0.4	24.0%
Zinc(II)	Cys	Sγ	2.3	0.2	100.0%
Zinc(II)	Glu	Oε1	2.0	0.3	100.0%
Zinc(II)	Glu	Oε2	2.8	0.4	71.9%
Zinc(II)	Glu	Oε2	4.1	0.5	28.1%
Zinc(II)	His	Nδ1	2.1	0.2	100.0%
Zinc(II)	His	Nε2	2.1	0.2	100.0%

Table S8 Distances computed for homodinuclear sites in the < 1.5 Å resolution range.

**Homodinuclear**			
**Metal**	**Peak center (Å)**	**Std (Å)**	**Contribution to the peak**
Sodium(I)	3.5	0.4	100.0%
Magnesium(II)	3.6	0.3	90.4%
Magnesium(II)	6.4	0.5	9.6%
Calcium(II)	3.9	0.3	76.6%
Calcium(II)	9.6	0.2	23.4%
Manganese	3.5	0.3	77.1%
Manganese	4.8	0.5	22.9%
Iron	3.3	0.3	100.0%
Nickel	3.6	0.2	94.9%
Nickel	5.1	0.0	5.1%
Copper	3.7	0.4	76.9%
Copper	6.1	0.3	23.1%
Zinc(II)	3.5	0.4	100.0%

Table S9 Distances computed for hetrodinuclear sites in the < 1.5 Å resolution range.

**Heterodinuclear**			
**Metal**	**Peak center (Å)**	**Std (Å)**	**Contribution to the peak**
Sodium(I)	4.8	0.5	56.3%
Sodium(I)	8.9	0.6	43.7%
Magnesium(II)	3.8	0.4	100.0%
Calcium(II)	4.4	0.4	82.7%
Calcium(II)	9.61	0.04	17.3%
Manganese	3.9	0.3	100.0%
Iron	2.9	0.3	74.0%
Iron	10.2	0.2	26.1%
Nickel	2.7	0.1	96.0%
Nickel	4.2	0.00	4.0%
Copper	5.0	0.01	53.2%
Copper	6.0	0.3	46.8%
Zinc(II)	3.7	0.3	54.1%
Zinc(II)	7.1	0.3	45.9%

Table S10 Distances computed for homodinuclear sites in the 1.5-2 Å resolution range.

**Homodinuclear**			
**Metal**	**Peak center (Å)**	**Std (Å)**	**Contribution to the peak**
Sodium(I)	3.5	0.4	100.0%
Potassium(I)	4.1	0.5	66.5%
Potassium(I)	7.7	0.1	33.5%
Magnesium(II)	3.6	0.3	90.1%
Magnesium(II)	6.4	0.5	9.9%
Calcium(II)	3.9	0.3	77.7%
Calcium(II)	9.6	0.2	22.3%
Manganese	3.6	0.3	100.0%
Iron	3.3	0.3	95.8%
Iron	5.1	0.4	4.2%
Nickel	3.6	0.2	89.5%
Nickel	5.2	0.3	10.5%
Copper	4.3	0.5	86.1%
Copper	5.9	0.6	13.9%
Zinc(II)	3.5	0.4	100.0%

Table S11 Distances computed for heterodinuclear sites in the 1.5-2 Å resolution range.

**Heterodinuclear**			
**Metal**	**Peak center (Å)**	**Std (Å)**	**Contribution to the peak**
Sodium(I)	3.9	0.4	77.3%
Sodium(I)	8.0	0.6	22.7%
Magnesium(II)	3.7	0.3	89.3%
Magnesium(II)	5.6	0.4	10.7%
Calcium(II)	4.2	0.2	89.7%
Calcium(II)	7.2	0.4	10.3%
Manganese	4.1	0.4	100.0%
Iron	3.7	0.3	67.0%
Iron	10.2	0.4	33.0%
Nickel	2.9	0.4	100.0%
Copper	4.9	0.4	33.1%
Copper	6.2	0.2	66.9%
Zinc(II)	3.8	0.3	67.9%
Zinc(II)	6.9	0.5	32.1%

Table S12 Distances computed for homodinuclear sites in the 2-2.5 Å resolution range.

**Homodinuclear**			
**Metal**	**Peak center (Å)**	**Std (Å)**	**Contribution to the peak**
Sodium(I)	3.9	0.5	100.0%
Magnesium(II)	3.7	0.4	90.3%
Magnesium(II)	6.5	0.6	9.7%
Calcium(II)	3.9	0.3	79.1%
Calcium(II)	9.8	0.2	20.9%
Manganese	3.3	0.3	86.2%
Manganese	4.8	0.5	13.8%
Iron	3.2	0.4	65.7%
Iron	4.0	0.4	34.3%
Nickel	3.6	0.3	88.6%
Nickel	5.4	0.4	11.5%
Copper	3.9	0.5	100.0%
Zinc(II)	3.5	0.3	100.0%

Table S13 Distances computed for heterodinuclear sites in the 2-2.5 Å resolution range.

**Heterodinuclear**			
**Metal**	**Peak center (Å)**	**Std (Å)**	**Contribution to the peak**
Sodium(I)	3.8	0.4	100.0%
Magnesium(II)	3.7	0.3	100.0%
Calcium(II)	4.2	0.2	100.0%
Manganese	3.4	0.3	19.8%
Manganese	4.2	0.4	80.2%
Iron	3.2	0.4	100.0%
Nickel	2.7	0.2	86.4%
Nickel	4.6	0.4	13.6%
Copper	4.7	0.3	49.4%
Copper	6.2	0.3	50.6%
Zinc(II)	3.8	0.3	65.3%
Zinc(II)	7.1	0.3	34.7%

Table S14 Distances computed for homodinuclear sites in the 2.5-3 Å resolution range.

**Homodinuclear**			
**Metal**	**Peak center (Å)**	**Std (Å)**	**Contribution to the peak**
Sodium(I)	3.7	0.4	85.2%
Sodium(I)	8.8	0.6	14.8%
Magnesium(II)	3.9	0.5	100.0%
Calcium(II)	4.0	0.5	88.0%
Calcium(II)	9.7	0.4	12.0%
Manganese	3.4	0.4	100.0%
Iron	3.1	0.3	65.1%
Iron	3.6	0.4	35%
Nickel	3.4	0.4	100.0%
Copper	3.1	0.5	13.8%
Copper	4.3	0.2	86.2%
Zinc(II)	3.4	0.3	100.0%

Table S15 Distances computed for heterodinuclear sites in the 2.5-3 Å resolution range.

**Heterodinuclear**			
**Metal**	**Peak center (Å)**	**Std (Å)**	**Contribution to the peak**
Sodium(I)	4.0	0.4	59.9%
Sodium(I)	12.1	0.2	40.1%
Magnesium(II)	3.7	0.4	100.0%
Calcium(II)	4.4	0.3	94.3%
Calcium(II)	6.0	0.5	5.7%
Manganese	4.5	0.4	100.0%
Iron	3.3	0.3	100.0%
Nickel	2.9	0.2	100.0%
Copper	4.8	0.2	67.7%
Copper	6.3	0.3	32.3%
Zinc(II)	3.8	0.4	70.4%
Zinc(II)	7.1	0.4	29.6%

Table S16 Percentage of water molecules interacting with each metal ion in mononuclear sites for theresolution range < 1.5 Å.

N° of water molecules	Sodium(I)	Potassium(I)	Magnesium(II)	Calcium(II)	Manganese	Iron	Nickel	Copper	Zinc(II)
0	18.8%	36.5%	24.4%	14.3%	13.5%	69.0%	74.0%	81.4%	78.4%
1	23.4%	26.5%	15.5%	28.3%	55.8%	25.6%	10.3%	13.4%	14.5%
2	24.7%	25.6%	18.3%	34.0%	13.5%	3.1%	6.3%	5.2%	4.9%
3	16.4%	6.2%	19.2%	11.2%	10.4%	2.2%	5.4%	/	1.9%
4	10.2%	2.4%	13.4%	7.8%	4.3%	/	4.0%	/	0.2%
5	6.3%	2.4%	9.1%	3.3%	2.5%	/	/	/	0.2%
6	0.3%	0.5%	0.2%	1.0%	/	/	/	/	0.1%
7	/	/	/	0.1%	/	/	/	/	/

Table S17 Number of sites and the computed distances for each metal subdivided according to the nuclearity.

Metal	Nuclearity	N° of sites	N° of distances
Sodium(I)	1.0	12016	36913
Sodium(I)	2.0	687	3018
Sodium(I)	3	233	1033
Potassium(I)	1	3519	12586
Potassium(I)	2	509	2595
Potassium(I)	1	3519	12586
Calcium(II)	1	18451	100604
Calcium(II)	2	2713	28907
Calcium(II)	3	682	13858
Magnesium(II)	1	20239	56947
Magnesium(II)	2	3582	17447
Magnesium(II)	3	879	6484
Manganese	1	3361	14481
Manganese	2	2308	20950
Manganese	3	140	1615
Iron	1	13495	26366
Iron	2	2404	15398
Iron	3	450	1754
Nickel	1	1935	6240
Nickel	2	264	1402
Copper	1	2249	7248
Copper	2	458	2146
Copper	3	229	1834
Zinc(II)	1	21462	78770
Zinc(II)	2	3063	22535
Zinc(II)	3	382	3938
